# Carbon dioxide responsiveness mitigates rice yield loss under high night temperature

**DOI:** 10.1093/plphys/kiab470

**Published:** 2021-10-13

**Authors:** Rajeev Nayan Bahuguna, Ashish Kumar Chaturvedi, Madan Pal, Chinnusamy Viswanathan, S V Krishna Jagadish, Ashwani Pareek

**Affiliations:** 1 Division of Plant Physiology, Indian Agricultural Research Institute, New Delhi 110012, India; 2 Centre for Advance Studies on Climate Change, Dr Rajendra Prasad Central Agricultural University, Samastipur 848125, India; 3 Land and Water Management Research Group, Centre for Water Resources Development and Management, Kozhikode 673571, India; 4 Department of Agronomy, Kansas State University, Manhattan, Kansas 66506, USA; 5 Department of Crop Physiology, University of Agricultural Sciences, Bengaluru 560065, India; 6 School of Life Sciences, Stress Physiology and Molecular Biology Laboratory, Jawaharlal Nehru University, New Delhi 110067, India

## Abstract

Increasing night-time temperatures are a major threat to sustaining global rice (*Oryza sativa* L.) production. A simultaneous increase in [CO_2_] will lead to an inevitable interaction between elevated [CO_2_] (e[CO_2_]) and high night temperature (HNT) under current and future climates. Here, we conducted field experiments to identify [CO_2_] responsiveness from a diverse *indica* panel comprising 194 genotypes under different planting geometries in 2016. Twenty-three different genotypes were tested under different planting geometries and e[CO_2_] using a free-air [CO_2_] enrichment facility in 2017. The most promising genotypes and positive and negative controls were tested under HNT and e[CO_2_] + HNT in 2018. [CO_2_] responsiveness, measured as a composite response index on different yield components, grain yield, and photosynthesis, revealed a strong relationship (*R*^2^ = 0.71) between low planting density and e[CO_2_]. The most promising genotypes revealed significantly lower (*P* < 0.001) impact of HNT in high [CO_2_] responsive (HCR) genotypes compared to the least [CO_2_] responsive genotype. [CO_2_] responsiveness was the major driver determining grain yield and related components in HCR genotypes with a negligible yield loss under HNT. A systematic investigation highlighted that active selection and breeding for [CO_2_] responsiveness can lead to maintained carbon balance and compensate for HNT-induced yield losses in rice and potentially other C_3_ crops under current and future warmer climates.

## Teaser

Active selection for carbon dioxide responsiveness in rice and other C_3_ crops can mitigate yield loss induced by high night temperature.

## Introduction

Global climate change is predominantly associated with rising [CO_2_] concentration and climate warming. Crop responses to these two environmental changes, both separately and in combination, vary significantly within and between crop species (Allen, 1991; Prasad et al., 2005; Ziska et al., 2012). Rice (*Oryza sativa* L.) is the most important cereal crop providing calories to >3.5 billion people worldwide (Muthayya et al., 2014). Being a C_3_ crop, it can directly benefit from elevated CO_2_ (e[CO_2_]) fertilization effect; however, a concomitant increase in ambient temperature is documented to negate the positive benefit of e[CO_2_] (Moya et al., 1998; Ziska et al., 2014; Chaturvedi et al., 2017). By disaggregating the global mean temperature increase, a relatively higher increase in minimum night temperature compared with maximum day temperature has been reported by several studies (Dai et al., 2001; Alexander et al., 2006; Sillmann et al., 2013a, 2013b). High night temperature (HNT) increases carbon loss from night respiration and reduces the activity of source–sink enzymes resulting in lower biomass, poor grain filling, and reduced grain weight (Bahuguna et al., 2017; Shi et al., 2013, 2017). Negative impacts of HNT on reducing rice yield and grain quality have been documented at the farm and regional scales (Peng et al., 2004; Welch et al., 2010), resulting in substantial economic losses (Lyman et al., 2013).

There are no escape or avoidance mechanisms reported in rice under HNT stress, which are otherwise effective strategies under high day temperature (HDT) stress (Ishimaru et al., 2010; Hirabayashi et al., 2015; Bahuguna et al., 2014, 2015). Thus, developing cultivars resilient to HNT is considered a viable option. Based on the differential physiological responses of rice to HDT and HNT (Jagadish et al., 2015), addressing carbon imbalance (photosynthesis versus respiration) and altered starch metabolism (Sadok and Jagadish, 2020) under HNT requires a different research strategy to sustain rice productivity under warmer nights. In addition, under current and future climates, night temperatures and [CO_2_] levels are predicted to increase simultaneously, hence rice would inevitably be exposed to e[CO_2_] and HNT environments. Enhancing crop’s ability to meet the increased carbon demand due to higher night respiration is hypothesized to minimize the negative impact of HNT on grain yield and quality (Impa et al., 2020). However, the effectiveness of increased responsiveness of crops to [CO_2_], as a means to meet the carbon demand under warmer nights and the e[CO_2_] + HNT interactions have not been investigated in any crop.

Exploring genetic diversity is suggested as a promising approach to identify potential tolerant donors, quantitative trait locus (QTL), and genes that can enhance stress tolerance and sustain yield gains under changing climate (Zhang et al., 2017). Rice responses to e[CO_2_] and the beneficial impact of e[CO_2_] are well documented, but knowledge generated so far has involved very few genotypes, with many studies dealing with one to eight genotypes (Sakai et al., 2019; Lv et al., 2020). Genetic diversity in rice for growth- and yield-related traits under e[CO_2_], hereafter referred to as [CO_2_] responsiveness, has not been explored primarily due to the limitation of space in [CO_2_] enrichment facilities and the significant cost of operation (Kim et al., 2003; Ainsworth and Long, 2005). Nevertheless, active selection aimed at identifying cultivars with improved [CO_2_] responsiveness could be exploited as an effective way to enhance yield under future [CO_2_] rich environments (Ziska et al., 2012). Altered planting geometry termed as low planting density (LPD) has been proposed as a surrogate method to explore phenotypic plasticity in rice for [CO_2_] responsiveness, which is demonstrated to significantly correlate with rice response under e[CO_2_] (Shimono, 2011; Shimono et al., 2014). Previous studies have mostly relied on phenotypic variation observed for individual yield components such as panicle number, panicle weight under LPD, and e[CO_2_] (Shimono, 2011; Shimono et al., 2014; Kikuchi et al., 2017), while yield is determined by a combination of multiple traits, which interact and have inherent tradeoffs. In addition, physiological traits such as photosynthesis that is directly influenced by [CO_2_] have not been considered while identifying [CO_2_]-responsive rice genotypes under different planting geometry studies. Considering that different planting geometry affects the physiology and [CO_2_] response (Kikuchi et al., 2017), with no individual phenotypic trait capturing the entire proportion of [CO_2_] responsiveness (Dingkuhn et al., 2020), aggregating phenotyping plasticity, and deriving a composite response index (CRI) could increase the efficiency in identifying high [CO_2_]-responsive (HCR) donors.

**Figure 1:**
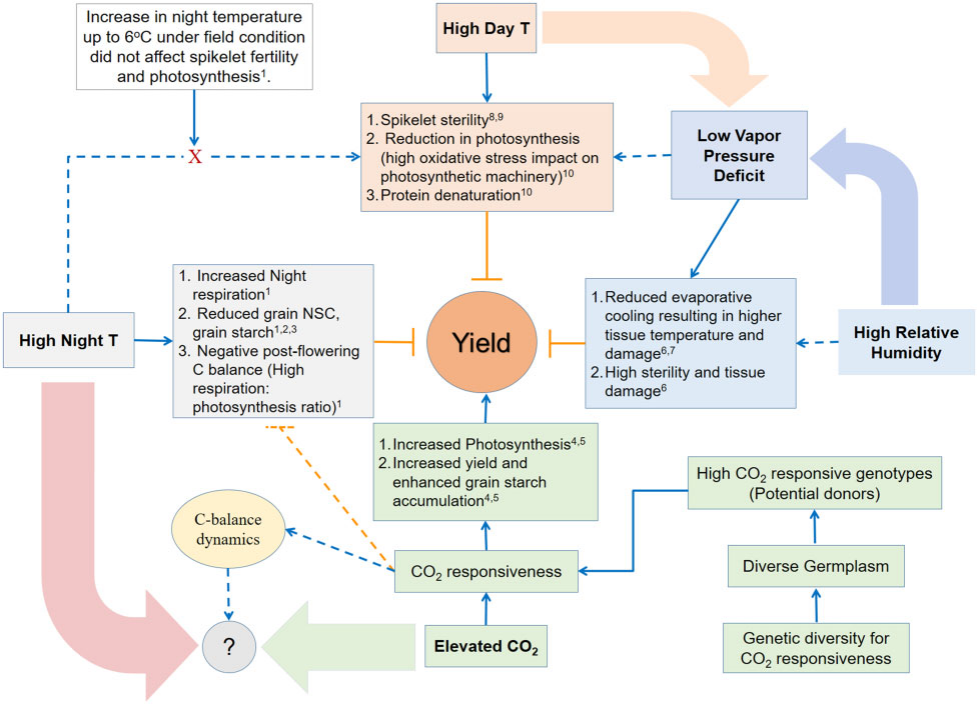
Schematic diagram illustrating key traits affected by HDT, HNT, RH, and e[CO_2_] and subsequently their impact on rice yield. A well-documented interaction of HDT and humidity (vapor pressure deficit) determines severity of day time temperature. On the other hand, HNT majorly affects night respiration and starch metabolism in the developing grains. Effect of e[CO_2_] provides additional substrate to photosynthesis and subsequently enhances assimilate production. Elevated [CO_2_] and HNT converse at C-balance dynamics due to (1) direct (positive) effect of e[CO_2_] on photosynthesis (C-gain) and carbohydrate metabolism and (2) direct (negative) effect of HNT on respiration (C-loss) and carbohydrate metabolism. Exploring and integrating genetic diversity for [CO_2_] responsiveness can help maintain a positive carbon balance and compensate stress-induced C loss. The blue solid arrows indicate direct effect, blue dotted arrows indicate plausible effect, orange solid arrows indicate direct negative effect, and orange dotted arrow indicate plausible negative effect on the physiological processes (traits). “?” indicates current research gap. (C, carbon; T, temperature; NSC, nonstructural carbohydrate; References: ^1^Bahuguna et al., 2017; ^2^Shi et al., 2017; ^3^Shi et al., 2013; ^4^Chaturvedi et al., 2017; ^5^Leakey et al., 2009; ^6^Bahuguna et al., 2015; ^7^Sadok and Jagadish, 2020; ^8^Jagadish et al., 2007; ^9^Jagadish et al., 2010; ^10^Mittler et al., 2012).

Atmospheric [CO_2_] levels are predicted to reach ∼560 µmol mol^−1^ by 2,050 and ∼700 µmol mol^−1^ by 2,100 (Leakey et al., 2009; IPCC, 2014; Sekhar et al., 2020). The most recent IPCC 2021 Summary for Policymakers report (IPCC, 2021), incorporating more recent data reveal that in the longer term (2,081–2,100), a very likely increase in average global temperature to be as high as 5.7°C (best estimate of 4.4°C). Interestingly, effect of both HNT and e[CO_2_] on biomass and yield converge through their effects on carbon balance dynamics, but in opposite ways (Figure 1). While HNT causes additional loss of carbon through respiration (night respiration; Mohammad and Tarpley, 2009; Peraudeau et al., 2015a, 2015b; Bahuguna et al., 2017), affecting starch synthesis and accumulation in rice grains (Bahuguna et al., 2017; Shi et al., 2013, 2017), rise in [CO_2_] provides additional substrate to photosynthesis and thus fixes more carbon (Ziska and Teramura, 1992; Chen et al., 2014; Chaturvedi et al., 2017). Despite the opposing responses, [CO_2_] and HNT interactions have to be systematically evaluated considering their inevitable interaction under current and future climate, and the potential to breed for improved carbon balance in HNT sensitive C3 cereals. Hence, we hypothesized that rice cultivars having significantly higher [CO_2_] responsiveness could efficiently fix additional carbon available under current and future environments to augment loss of carbon (C) and minimize the negative impact of HNT (Figure 1). Field experiments were conducted over 3 years using field-based free-air [CO_2_] enrichment (FACE) and HNT tent facilities and involving an indica diversity panel (194 genotypes) to (1) capture [CO_2_] responsiveness in rice through CRI including growth, yield, and photosynthesis, by employing different planting geometries and (2) ascertain rice responses to HNT and e[CO_2_] + HNT conditions to quantify the potential of increased [CO_2_] responsiveness in alleviating the negative impact of HNT on rice yield losses.

## Results

### Environmental conditions

**Figure 2:**
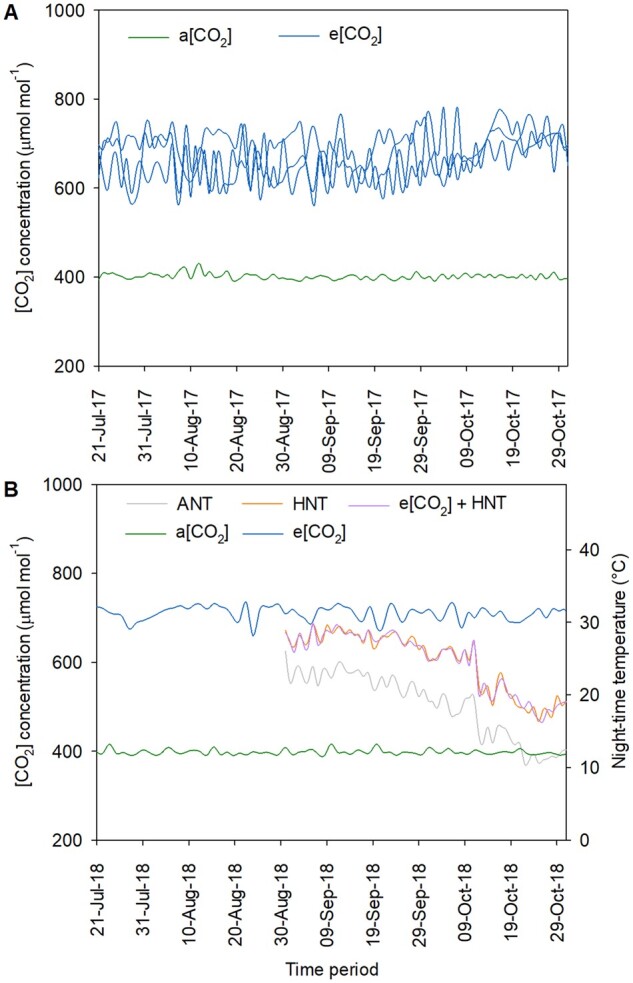
[CO_2_] and temperature in FACE rings and HNT tents. Environmental parameters showing daily ambient [CO_2_] concentration (green line) and e[CO_2_] concentration (blue lines) averaged across three independent FACE rings during 2017 (A); daily ambient [CO_2_] concentration (green line), and e[CO_2_] concentration (blue line) in night temperature tent during 2018; daily night time temperature under ambient (gray line), HNT tent (orange line), and HNT + e[CO_2_] (e[CO_2_] + HNT) tent (purple line) (B); e[CO_2_] exposure during 2017 and 2018 experiments was initiated at seven days after transplanting and continued until physiological maturity. HNT was started at panicle initiation and continued until physiological maturity.

Temperature, relative humidity (RH), and rainfall data were recorded during Kharif season (June–October) in 2016, 2017, and 2018. Environmental parameters were consistent and did not vary significantly during the experiment period across years (Supplemental Figure S1, A–C). Average mean day and night temperatures with standard deviation (SD) were 34.8°C (sd ± 2.9) and 22.4°C (sd ± 4.0) in 2016; 34.5°C (sd ± 2.8) and 23.7°C (sd ± 3.9) in 2017; and 34.2°C (sd ± 3.5) and 22.9°C (sd ± 4.8) in 2018, respectively. Average daily RH varied between 77.8 ± 11.9% and 74.3 ± 11.4% between 2016 and 2018. A normal rainfall pattern was observed during Kharif season with total average rainfall ranging between 839.8 and 1,156.9 mm during the experimental period in 2016, 2017, and 2018 (Supplemental Figure S1, A–C). Average [CO_2_] concentration in ambient condition was 401.2 (sd ± 6.6) µmol mol^−1^ during 2017 and 399.1 (sd ± 5.9) µmol mol^−1^ during 2018 (Figure 2, A and B). [CO_2_] treatment resulted in e[CO_2_] ranging between 662.9 (sd ± 43.9) and 687.7 (sd ± 47.2), averaged across the three FACE rings in 2017 (Figure 2A), while it was 709.9 (sd ± 15.5) in e[CO_2_] + HNT tent during 2018 (Figure 2B). HNT treatment increased air temperature by ∼5.6°C during the night (1800–0600 h) with an average night temperature of 27.7°C (sd ± 1.4) in HNT treatment and 27.6°C (sd ± 1.3) in e[CO_2_] + HNT tent, respectively (Figure 2B).

### Phenotypic plasticity of growth, yield, and yield components under LPD

**Figure 3:**
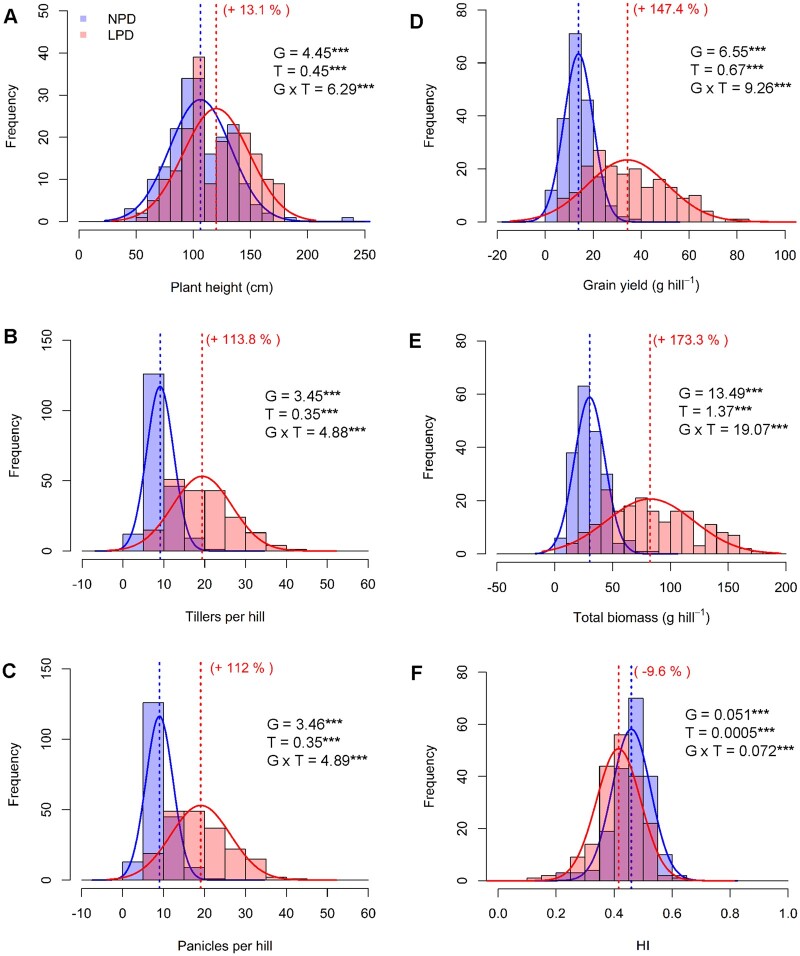
Phenotypic variation in yield components under NPD and LPD. Overlying histograms with normal distribution curves (NPD, blue line, bars; LPD, orange line, bars and overlap between treatments with the lower frequency value, intermediate pink bars) showing the phenotypic distribution of growth and yield components viz. plant height (A) tillers (B), panicles (C), grain yield (D), total biomass (E), and HI (F) among 194 rice genotypes in 2016. The vertical dotted lines in the histograms show population mean in NPD (blue) and LPD (orange) conditions, and values in parentheses represent the significant percentage change (+, increase; –, decrease) in LPD as compared to NPD. Levels of significance for genotype (G) and treatment (T) effects from analysis of variance (ANOVA) are given with Fisher’s least significant difference (LSD) value (*P* < 0.05). (Significance: ^***^P < 0.001).

In Experiment I, a significant genotype (G), treatment (T), and G × T (*P* < 0.001) effect was observed for growth, grain yield, and yield components (Supplemental Table S1). Grain yield recorded 147% increase while growth and yield components (plant height, number of tillers and panicles, and total biomass per hill) recorded 13%–173% increase under LPD compared to the normal planting density (NPD). In contrast, harvest index (HI) decreased by 10% under LPD as compared to NPD (Figure 3, A–F; Supplemental Table S1). A significant treatment effect (*P* < 0.001) on phenotypic plasticity was observed with growth, grain yield, and yield components under LPD with higher phenotypic variability in the population under LPD compared to the NPD (Figure 3, A–F).

### Phenotypic plasticity of photosynthesis and related traits under LPD

A significant genotype (G), treatment (T), and G × T (*P* < 0.001) effect was recorded for greenness index calculated as soil plant analysis development (SPAD) value, photosynthesis, and related gas exchange traits (Supplemental Table S1). SPAD value increased by 9% under LPD averaged across 191 rice genotypes (Supplemental Table S1; Supplemental Figure S2A). Phenotypic plasticity recorded for gas exchange traits showed the highest plasticity for stomatal conductance (*g_s_*) followed by photosynthesis (*A*) and transpiration (*E*) recording 41%, 26%, and 24% increase under LPD, compared to NPD, respectively (Supplemental Figure S3, A–C; Supplemental Table S1). Phenotypic variability for photosynthesis and related traits was significantly higher under LPD compared to NPD (Supplemental Figure S4, A–C).

### Composite phenotypic plasticity under LPD

Genotypes were categorized as low to high responsive under LPD based on their response index (RI) calculated individually for growth, grain yield, and yield components (Supplemental Figure S5, A–E). A composite response index (CRI) was calculated as the sum of individual response indices including growth, yield, yield components, and photosynthesis (*A*). Based on CRI values, all genotypes were divided into three equal parts and categorized as low (CRI ≤ 0.83), moderate (CRI ≥ 0.84 to <1.20), and high (CRI ≥ 1.21) phenotypic plasticity group. Furthermore, based on similar phenology, i.e. days to heading, 23 genotypes were selected with 13 genotypes from high CRI, 4 from moderate, and 6 from low phenotypic plasticity groups (Supplemental Figure S6). These 23 genotypes were used in experiment II to validate the [CO_2_] response under LPD, NPD, and e[CO_2_] treatments.

### Comparative response of yield and yield components under LPD and e[CO_2_]

In Experiment II, 23 rice genotypes grown under NPD, LPD, and e[CO_2_] showed significant genotype (*P* < 0.001) and treatment (*P* < 0.001) effects for grain yield and yield components (Table 1). Treatment effect was non-significant for plant height and HI. However, a significant G × T effect was noted for plant height (*P* < 0.001), tillers, and panicles per hill and HI (*P* < 0.01) and thousand grain weight (TGW) (*P* < 0.001). G × T effect was non-significant for grain yield and total biomass (Table 1; Supplemental Table S2). Both LPD and e[CO_2_] treatment significantly increased tiller number (72% and 43%), panicle number (71% and 42%), grain yield (44% and 23%), total biomass (49% and 25%), and TGW (3.5% and 5.5%), compared to NPD, respectively (Table 1). Conversely, HI was reduced by 2.8% and 0.8% under LPD and e[CO_2_] compared to NPD, respectively (Table 1), similar to 194 genotypes in Experiment I (Figure 3). The phenotypic variation observed for growth, grain yield, and yield components was highest in LPD followed by e[CO_2_], compared to NPD (Table 1).

**Table 1:** Descriptive statistics and the significance (Fischer’s test summary) for genotype (G), treatment (T) and their interactions (G × T) for different growth, yield and physiological traits in a diverse set of rice genotypes (*n* = 23) grown under NPD, LPD, and e[CO_2_] in 2017

Traits	NPD			LPD			e[CO_2_]			% C	% C	*P* value (Fischer’s test)		
	Mean ± sd	Minimum	Maximum	Mean ± sd	Minimum	Maximum	Mean ± sd	Minimum	Maximum	LPD	FACE	G	T	G × T
Yield traits														
Plant height	114.78 ± 21.29	66.50	145.20	115.13 ± 15.35	81.98	139.80	114.97 ± 14.26	79.92	134.92	0.30	0.16	<0.001	**0.103**	<.001
Tillers hill^−1^	14.94 ± 4.59	9.70	26.86	25.75 ± 7.83	14.60	43.80	21.36 ± 6.17	13.00	37.40	72.31	42.92	<0.001	<0.001	0.004
Panicles hill^−1^	14.93 ± 4.61	9.70	26.86	25.45 ± 7.88	14.00	43.80	21.19 ± 6.09	12.80	36.80	70.52	41.96	<0.001	<0.001	0.002
Total biomass hill^−1^	60.49 ± 12.46	40.06	78.72	90.13 ± 18.59	58.95	118.07	75.85 ± 14.54	56.78	100.56	49.00	25.39	<0.001	<0.001	**0.894**
Grain yield hill^−1^	29.10 ± 7.13	19.01	41.52	41.82 ± 8.47	26.59	56.45	35.92 ± 7.47	24.02	51.40	43.69	23.42	<0.001	<0.001	**0.988**
TGW (g)	22.36 ± 3.87	17.07	30.19	23.14 ± 4.03	17.80	32.17	23.59 ± 3.96	17.66	32.47	3.49	5.49	<0.001	<0.001	<.001
HI	0.48 ± 0.05	0.35	0.56	0.47 ± 0.05	0.37	0.59	0.48 ± 0.04	0.38	0.56	−2.76	−0.82	<0.001	**0.373**	0.007
Physiological traits														
SPAD	37.67 ± 2.11	33.27	40.80	41.23 ± 2.54	36.80	45.70	38.77 ± 2.38	34.30	42.50	9.47	2.92	<0.001	<.001	**0.327**
*A* (µmol m^−2^ s^−1^)	18.14 ± 4.03	12.13	26.60	24.27 ± 3.95	14.75	31.16	27.49 ± 4.04	18.57	33.66	33.83	51.57	<0.001	<0.001	<.001
*g_s_* (mol m^−2^ s^−1^)	0.32 ± 0.12	0.14	0.58	0.56 ± 0.10	0.28	0.77	0.39 ± 0.11	0.20	0.55	77.01	22.38	<0.001	<0.001	<.001
*E* (mmol m^−2^ s^−1^)	8.35 ± 1.83	4.84	12.28	9.48 ± 1.41	7.48	12.69	8.14 ± 1.56	5.65	12.52	13.50	−2.49	<0.001	<0.001	<.001

% C: % change (+: increase or –: decrease) over NPD, Bold *P*-values are not statistically significant (*P* ≥ 0.05) (*A*, photosynthesis; *g*_s_, stomatal conductance; *E*, transpiration).

### Comparative response of photosynthetic traits under LPD and e[CO_2_]

**Figure 4:**
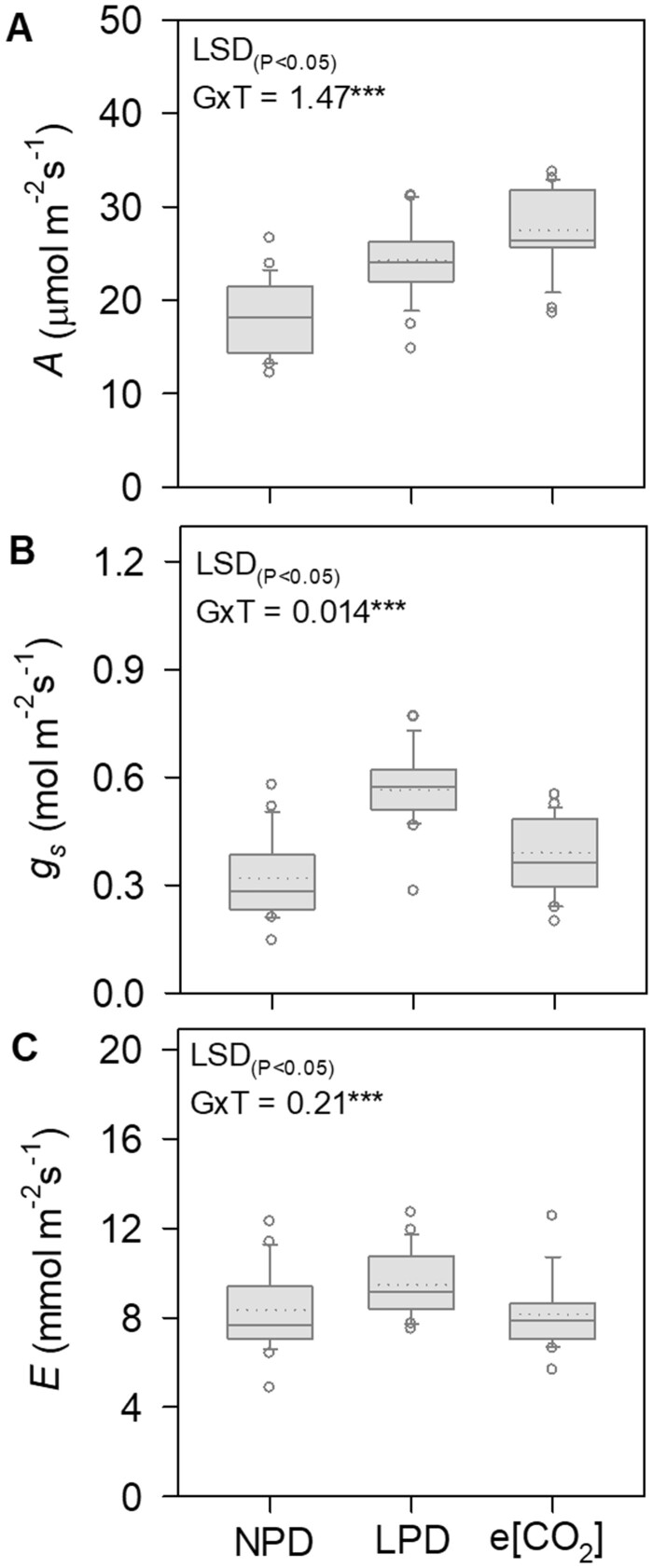
Phenotypic variation in gas exchange traits under different planting densities and e[CO_2_]. Box-plot showing phenotypic variation of leaf photosynthetic rate (*A,* A), stomatal conductance (*g_s_*, B) and transpiration rate (*E*, C) in 23 rice genotypes under NPD, LPD, and e[CO_2_] during 2017. Inside box-plot, the solid and dotted lines represent the median and the mean of the population, respectively. Box edges represent upper and lower quantiles, and whiskers are 1.5× the quantile of the data. Outliers are shown as open circles. Levels of significance for genotype (G) and treatment (T) effects from ANOVA are given with Fisher’s LSD value (*P* < 0.05). (Significance: ^***^P < 0.001).

A significant genotype (G), treatment (T), and G × T (*P* < 0.001) effect was observed for photosynthesis and related traits in Experiment II (Table 1). Greenness index recorded significant genotype and treatment (*P* < 0.001) effect with 9.5% and 2.9% increase in mean SPAD value under LPD and e[CO_2_] compared to NPD, respectively (Table 1; Supplemental Figure S2B). Elevated [CO_2_] treatment increased *A* by 52% ranging 19–34 µmol m^−2^ s^−1^ between the genotypes compared to NPD (12–27 µmol m^−2^ s^−1^) (Figure 4A and Table 1). Conversely, *g_s_* recorded 22% increase while *E* recorded 2.5% decrease under e[CO_2_] compared to NPD (Figure 4, B and C; Table 1). Similarly, genotypes grown in LPD recorded 13%–77% increase in photosynthesis and related traits compared to the NPD (Table 1).

### PCA of yield components and photosynthetic traits under LPD and e[CO_2_]

In Experiment I, the first two principal components (PCs) cumulatively explained >63% and >59% of the total phenotypic variation among different variables in NPD and LPD, respectively (Supplemental Figure S7, A and B). The phenotypic variation in the PC1 was mostly explained by total biomass, grain yield, panicles, and tillers across the treatments. Conversely, physiological parameters including *A*, *g_s_,* and *E* expressed most of the phenotypic variation in PC2 across the treatments (Supplemental Figure S7, A and B). In Experiment II, the two PCs cumulatively explained >62%, >50%, and >61% of the total phenotypic variation in NPD, LPD, and e[CO_2_], respectively (Supplemental Figure S7, C–E). Within NPD treatment, tillers and panicles accounted for most of the phenotypic variation in PC1 while total biomass, *A*, *g_s_,* and grain yield expressed most of the phenotypic variation in PC2 (Supplemental Figure S7C). On the other hand, in LPD, tillers, panicles, grain yield, and total biomass in PC1 and *A*, SPAD, and *g_s_* accounted for most of the phenotypic variation in PC2 (Supplemental Figure S7D). Under e[CO_2_], the most of phenotypic variation was expressed by panicles, tillers, grain yield, and *E* in PC1 while *g_s_*, *A*, and total biomass in PC2 (Supplemental Figure S7E).

### Validation of CO_2_ responsiveness under e[CO_2_]

**Figure 5:**
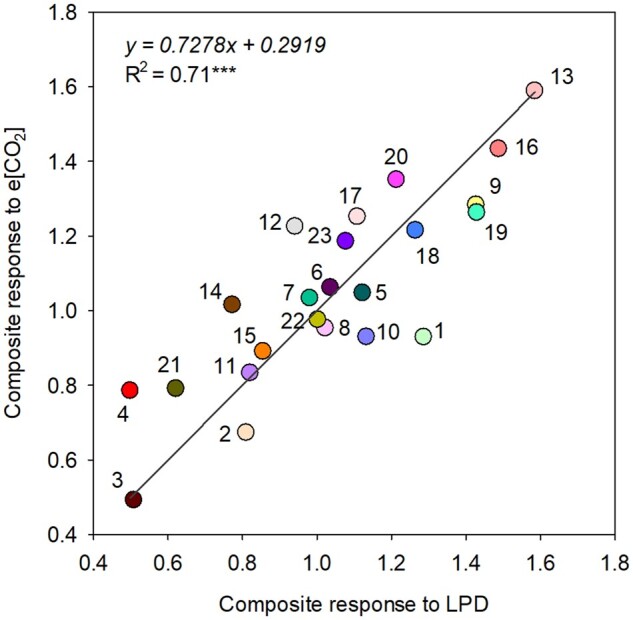
Relationship between the composite response of 23 rice genotypes to LPD and e[CO_2_] during 2017. Responsiveness of grain yield, yield components (tillers hill^−1^, panicles hill^−1^, grain yield hill^−1^, total biomass hill^−1^, and TGW), and photosynthesis were calculated individually for LPD and e[CO_2_] and then averaged to get a composite response under respective treatment. Each circle represents a genotype indicated with a number. (Significance for linear regression analysis: ^***^P < 0.001; 1, DZ78; 2, SML 242; 3, AI-CHIAO-HONG; 4, BYAKKOKU Y 5006 SELN; 5, CHAMPA TONG 54; 6, CHINESE; 7, DAWEBYAN; 8, DJ 123; 9, ECIA76-S89-1;10, GHARIB; 11, HALWA GOSE RED; 12, IRGA 409; 13, JC 117; 14, KARKATI 87; 15, KIANG-CHOU-CHIU; 16, MTU9; 17, MUDGO; 18, PEH-KUH; 19, PTB 30; 20, SURJAMKUHI; 21, TIA BURA; 22, YODANYA; and 23, RONDO).

In Experiment II, a significantly (*P* < 0.001) strong relationship (*R*^2^ = 0.71) was observed for composite response with LPD and e[CO_2_] (Figure 5). Composite response comprised phenotypic plasticity observed for grain yield and yield components (tillers, panicles, total biomass, and TGW) and photosynthesis with LPD and e[CO_2_]. Phenotypes included for composite response individually recorded a significantly (*P* < 0.01–0.001) strong relationship between LPD and e[CO_2_] on tillers (*R*^2^ = 0.82), panicles (*R*^2^ = 0.82), TGW (*R*^2^ = 0.33), total biomass (*R*^2^ = 0.58), grain yield (*R*^2^ = 0.45), and photosynthesis (*R*^2^ = 0.88) (Supplemental Figure S8). Based on the composite response, rice genotypes JC 117 and MTU9 were the most CO_2_ responsive and AI-CHIAO-HONG was the least responsive genotype across the treatments (Figure 5).

### Rice response to HNT and e[CO_2_] + HNT

In Experiment III, a contrasting set of rice genotypes tested under HNT and e[CO_2_] + HNT showed a significant genotype (G), treatment (T), and G × T (*P* < 0.001) effect on plant height, grain yield, and yield components (Table 2). Effect of HNT and e[CO_2_] + HNT on *A* varied from −0.6% to −15.3% and 5% to 92%, respectively, as compared to the control (Supplemental Figure S9A). Although treatment effect was non-significant, a significant G × T effect (*P* < 0.05 to <0.001) was observed for *g_s_* and *E* (Supplemental Figure S9, B and C; Supplemental Table S3). Night respiration (*R_N_*) increased significantly (*P* < 0.001) under HNT across the genotypes, where AI-CHIAO-HONG recorded a maximum increase (99.7%), and NL44 recorded the lowest (17.9%) *R_N_* compared to control conditions (Supplemental Figure S10A). Except the sensitive AI-CHIAO-HONG, there was no significant difference in *R_N_* under e[CO_2_] + HNT as compared to control across the genotypes (Supplemental Figure S10A; Supplemental Table S3). Conversely, HNT significantly (*P* < 0.001) increased *R_N_*/*A* ratio (19%–136%) across the genotypes in comparison to control, where maximum *R_N_*/*A* ratio was recorded for AI-CHIAO-HONG and MTU9 (0.051). However, *R_N_*/*A* ratio under e[CO_2_] + HNT were similar or lower (MTU9) than control across the genotypes except AI-CHIAO-HONG, which recorded the highest *R_N_*/*A* ratio (0.041) (Supplemental Figure S10B).

**Table 2:** Effect of HNT and e[CO_2_] + HNT interaction on growth, grain yield, and yield components of rice cultivars viz of rice cultivars viz. NL44 (heat-tolerant check), AL-CHIAO-HONG (LCR), ECIA76-S89-1, JC 117, MTU9 (HCR) during 2018. Each data represent mean of three replicates ± standard error of means

Genotype	Treatments	Plant height (cm)	Tillers hill^−1^	Panicles hill^−1^	Main panicle weight (g)	Grain yield (g hill^−1^)	Total biomass (g hill^−1^)	TGW (g)	HI
NL44	Control	104 ± 0.5	11 ± 0.3	10 ± 0.3	4.93 ± 0.10	31.4 ± 1.6	75.5 ± 3.4	22.38 ± 0.11	0.42 ± 0.010
	HNT	107 ± 0.9	10 ± 0.3	10 ± 0.3	4.64 ± 0.10	28.2 ± 1.0	67.5 ± 1.9	21.76 ± 0.03	0.42 ± 0.008
	e[CO_2_] + HNT	111 ± 0.6	14 ± 0.3	14 ± 0.3	4.89 ± 0.12	30.2 ± 1.3	77.8 ± 2.9	22.39 ± 0.07	0.39 ± 0.005
AI-CHIAO-HONG	Control	121 ± 0.6	23 ± 0.8	22 ± 0.8	3.54 ± 0.07	31.5 ± 1.1	72.6 ± 2.1	21.91 ± 0.05	0.43 ± 0.007
	HNT	112 ± 0.5	21 ± 0.2	20 ± 0.2	2.91 ± 0.07	21.4 ± 0.6	55.5 ± 1.2	20.03 ± 0.13	0.39 ± 0.011
	e[CO_2_] + HNT	117 ± 0.9	26 ± 1.1	25 ± 1.1	3.12 ± 0.07	25.3 ± 0.9	59.8 ± 2.1	21.35 ± 0.06	0.43 ± 0.009
ECIA76-S89-1	Control	103 ± 1.5	11 ± 0.4	11 ± 0.4	3.74 ± 0.10	19.3 ± 0.9	46.4 ± 1.9	19.72 ± 0.05	0.41 ± 0.008
	HNT	101 ± 0.7	10 ± 0.4	10 ± 0.4	3.49 ± 0.07	17.0 ± 0.6	41.6 ± 1.2	19.22 ± 0.05	0.41 ± 0.006
	e[CO_2_] + HNT	104 ± 0.4	19 ± 0.4	19 ± 0.4	4.13 ± 0.09	25.4 ± 1.0	58.5 ± 1.5	20.56 ± 0.14	0.43 ± 0.009
JC 117	Control	141 ± 0.7	13 ± 0.4	13 ± 0.3	3.38 ± 0.13	22.7 ± 1.1	52.4 ± 1.5	22.85 ± 0.04	0.43 ± 0.013
	HNT	145 ± 0.7	13 ± 0.3	13 ± 0.3	3.12 ± 0.11	20.1 ± 0.8	48.1 ± 1.3	22.08 ± 0.03	0.42 ± 0.010
	e[CO_2_] + HNT	142 ± 0.6	21 ± 0.3	21 ± 0.3	3.72 ± 0.10	29.4 ± 1.5	68.5 ± 3.0	23.76 ± 0.08	0.43 ± 0.009
MTU9	Control	137 ± 1.0	11 ± 0.3	11 ± 0.3	4.99 ± 0.10	25.9 ± 0.9	65.8 ± 1.6	28.85 ± 0.04	0.39 ± 0.009
	HNT	132 ± 0.7	10 ± 0.2	10 ± 0.2	4.56 ± 0.10	22.3 ± 0.9	58.1 ± 1.7	27.78 ± 0.09	0.38 ± 0.009
	e[CO_2_] + HNT	133 ± 0.6	17 ± 0.4	17 ± 0.4	5.35 ± 0.14	30.1 ± 1.5	77.3 ± 3.1	29.62 ± 0.12	0.39 ± 0.007
LSD (*P* < 0.05)	Genotype (G)	1.26^***^	0.75^***^	0.73^***^	0.16^***^	1.72^***^	3.47^***^	0.13^***^	0.014^***^
	Treatment (T)	0.98^*^	0.58^***^	0.56^***^	0.12^***^	1.33^***^	2.68^***^	0.10^***^	0.011^*^
	G × T	2.19^***^	1.29^***^	1.26^***^	0.27^***^	2.98^***^	6.00^***^	0.23^***^	0.025^**^

(Probability values of the effects of genotypes (G), treatment (T) and their interaction (G *×* T) for all the traits measured by ANOVA; significance,

*** *P* < 0.001;

** *P* < 0.01;

* *P* < 0.05).

HNT significantly (*P* < 0.001) reduced the number of tillers and panicles (2%–11%) across the genotypes compared to the control (Table 2). AI-CHIAO-HONG recorded the highest reduction (18%) in the main panicle weight under HNT followed by MTU9 (9%), JC 117 (8%), ECIA76-S89-1 (7%), and NL44 (6%), compared to their respective control. Moreover, grain yield was reduced by 10%–32% under HNT compared to the control, where AI-CHIAO-HONG recorded the highest and NL44 lowest reduction, respectively (Table 2). Similarly, reduction in total biomass and TGW ranged between 8%–24% and 3%–9%, respectively, across the genotypes with the highest reduction noted for AI-CHIAO-HONG compared to the control. HNT reduced HI (1%–11%) across the genotypes, where AI-CHIAO-HONG recorded maximum reduction compared to control (Table 2).

Phenotypic response to e[CO_2_] + HNT varied considerably across the different yield components. All the genotypes recorded an increase in number of tillers (13%–74%) and panicles (13%–73%) under e[CO_2_] + HNT compared to the control. However, AI-CHIAO-HONG recorded lower main panicle weight (12%), grain yield (20%), total biomass (18%), and TGW (3%) under e[CO_2_] + HNT compared to the control, following a similar pattern when exposed to HNT alone. Conversely, all genotypes recorded higher tillers and panicles (23%–97%), main panicle weight (5%–19%), grain yield (7%–49%), total biomass (8%–42%), and 1,000 grain weight (3%–8%) under e[CO_2_] + HNT as compared to HNT, where ECIA76-S89-1, JC 117, and MTU9 recorded significantly higher gain in grain yield and yield components, respectively (Table 2). HI changed significantly (*P* < 0.05) under e[CO_2_]  + HNT as compared to control (−7% to 4%) and HNT (−7% to 10%) (Table 2).

## Discussion

In this study, we demonstrate that [CO_2_] responsiveness could be a potential breeding target to improve rice yield under HNT. We have screened diverse rice germplasm for [CO_2_] responsiveness by using planting geometry and e[CO_2_] conditions. A contrasting set of genotypes were then tested for their response under HNT and e[CO_2_] + HNT interaction. The findings from our study are discussed below.

### Photosynthesis and agronomic plasticity under LPD captures [CO_2_] responsiveness

Despite the existence of a wide genetic diversity in C_3_ crops for [CO_2_] responsiveness, this has not been utilized in breeding to enhance crop yield under rising [CO_2_] environments, majorly due to the lack of potential donors and poor understanding about traits associated with [CO_2_] responsiveness (Ziska et al., 2012; Dingkuhn et al., 2020). Conclusions from [CO_2_] enrichment studies have strongly indicated that an increase in atmospheric [CO_2_] would substantially increase crop yield through active selection or development of [CO_2_]-responsive cultivars (Ziska et al., 2012). However, systematic attempts to exploit [CO_2_] responsiveness and associated physiological responses that lead to yield improvement are lacking. Limitation in space and accessibility to FACE and other [CO_2_] enrichment facilities under field conditions are major bottlenecks to characterize diverse set of genotypes under e[CO_2_]. Shimono (2011) and Shimono et al. (2014) reported LPD as a surrogate method to screen a diverse set of rice genotypes for [CO_2_] responsiveness. The phenotypic plasticity captured through LPD in terms of growth and yield under enhanced resources reflected plant response under realistic e[CO_2_] condition. In addition, earlier studies (Shimono, 2011; Shimono et al., 2014; Kumagai et al., 2015, 2016; Kikuchi et al., 2017) using LPD for [CO_2_] responsiveness have successfully validated the LPD to actual [CO_2_] response under [CO_2_] enrichment facilities in different C3 crops (rice, wheat, and soybeans), demonstrating a strong relationship in crops grown under different management and environmental conditions. Thus, in the first step, responses in 194 genotypes were overwhelming under LPD with significant phenotypic plasticity recorded for growth, grain yield, and yield components (Figure 3; Supplemental Figure S7; Supplemental Table S1) and physiological traits including photosynthesis (*A*), stomatal conductance (*g_s_*), and transpiration (*E*) (Supplemental Figures S3, S4, and S7).

Higher radiation reaching different positions along the canopy due to lesser overlap of leaves under LPD would help increase photosynthesis and associated gas exchange traits (Shimono et al., 2014, 2019). Moreover, the availability of more nutrients per unit area could translate to additional nutrient supply to aboveground parts, which has been documented to influence photosynthesis and carbon assimilation in plants (Ainsworth and Long, 2005). Variable responses have been reported on the effect of planting density on photosynthesis and associated gas exchange traits (Papadopoulos and Ormrod, 1988; Fang et al., 2018; Huang et al., 2021; Yan et al., 2021). A decrease in photosynthesis, stomatal density, stomatal conductance, and transpiration rate with increase in planting density has been observed across crop species including wheat and maize (Yan et al., 2017; Fang et al., 2018; Wei et al., 2020; Yan et al., 2021). This negative effect of reduced spacing on photosynthesis has been associated with leaf orientation, position, canopy structure, and crop growth stage (Papadopoulos and Ormrod, 1988; Wei et al., 2020; Yan et al., 2021). However, this systematic study in rice comprising 194 genotypes quantifies the effect of LPD on photosynthesis and associated gas exchange traits. Negative regulation of photosynthesis due to sink limitation is well documented (Paul and Foyer, 2001). Moreover, the rate of photosynthesis is strongly regulated by the sink size and strength (Dingkuhn et al., 2020). In our study, LPD significantly increased the number of tiller and panicles, which represents sink for vegetative and reproductive/grain-filling stage, respectively. Thus, a larger sink would have allowed for increased storage of photosynthates, supported by higher rate of photosynthesis (Dingkuhn et al., 2020). However, further research would help to unravel mechanisms on how LPD improves rate of photosynthesis and other gas exchange traits in rice.

In Experiment II, a selected set of 23 genotypes with varying phenotypic plasticity for growth, grain yield, yield components, and photosynthesis were validated under realistic e[CO_2_] condition (Table 1). Physiological traits such as *g_s_*, *E*, and greenness index had a contrasting response between LPD and e[CO_2_], and thus these traits were excluded as screening criteria under LPD (Figure 4 and Table 1; Supplemental Figure S2). A direct impact of e[CO_2_] on stomatal biology leading to stomatal closure and reducing transpiration is well documented (Long et al., 2004), while dilution of tissue nitrogen under e[CO_2_] is in line with the observed reduction in greenness index (Wang et al., 2015). Nevertheless, despite lower *g_s_* under e[CO_2_], phenotypic response of *A* showed significant similarity under LPD and e[CO_2_] (*R*^2^ = 0.74; *P* < 0.001; Supplemental Figure S11A), and phenotypic plasticity of *A* under LPD and e[CO_2_] (*R*^2^ = 0.88, *P* < 0.001; Supplemental Figure S11B). Besides contrasting responses of *g_s_*, *E*, and greenness index, all the yield-determining traits including photosynthesis recorded a significant increase under both LPD and e[CO_2_] (Table 1), validating LPD as an effective surrogate for screening large set of diverse genotypes for [CO_2_] responsiveness.

### CRI a robust measure of [CO_2_] responsiveness

Previous studies have estimated genetic variation for [CO_2_] responsiveness with a limited number of genotypes (Shimono, 2011; Shimono et al., 2014; Kumagai et al., 2015). Only recently, a large-scale genetic screening was done in rice for [CO_2_] responsiveness using the PRAY and MAGIC indica population, comprising 452 accessions (Kikuchi et al., 2017). In these studies, LPD is used to provide a resource-rich environment (light and nutrients) to estimate phenotypic plasticity for growth and yield traits of a genotype. However, phenotypic plasticity among traits, such as vegetative biomass, number of tillers, number of panicles, etc., possess inherent tradeoffs, suggesting that higher plasticity of an individual trait may not translate to higher yield per se (Ao et al., 2010; Li et al., 2019). In addition, grain yield is a complex trait determined by different growth and yield components, with these components influenced by strong genotype by environment interactions (Liang et al., 2015). Conversely, physiological traits such as photosynthesis are directly linked to [CO_2_] response and show linear increase with light and [CO_2_] under optimum range (Long et al., 2006). However, increase in photosynthesis too may not result in similar linear quantitative gain in yield due to its dynamic regulation by environment, sink limitation and transition (Dingkuhn et al., 2020). Hence, observing phenotypic plasticity under LPD with limited number of traits, independently, may not fully capture the benefits and tradeoffs from LPD or e[CO_2_] conditions on grain yield. Our findings provide support to a composite response approach by integrating growth, yield, and photosynthesis for screening genotypes under LPD and validating their response under e[CO_2_] as a means to eliminate potential tradeoffs compared to individual trait/s response approach. In addition, a CRI covering phenotypic plasticity involving multiple traits across LPD and e[CO_2_] enhanced the robustness of identifying potential [CO_2_]-responsive donors, with significantly strong relationship (*R*^2^ = 0.71, *P* < 0.001; Figure 5).

### Higher [CO_2_] responsiveness ameliorated HNT induced rice yield loss

**Figure 6:**
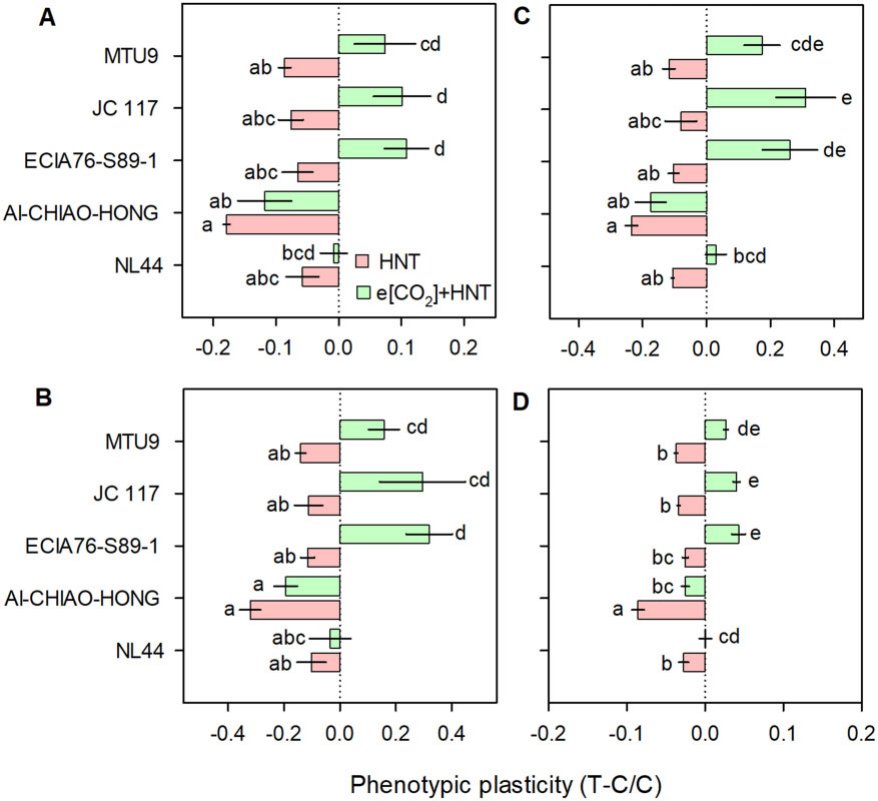
Summary of phenotypic variation in yield components under HNT and e[CO_2_] + HNT. Phenotypic plasticity of yield traits viz. main panicle weight (A), grain yield (B), total biomass (C), and TGW (D) in rice genotypes MTU9, JC 117, ECIA76-S89-1 (HCR), AI-CHIAO-HONG (LCR) and NL44 (HST) under HNT (red columns) and e[CO_2_] + HNT combination (green columns) compared to their respective controls (ambient temperature and ambient [CO_2_]). Phenotypic plasticity shows relative change between control (C) and treatment (T). Each horizontal column represents mean of five replicates. Bars indicate ± se (standard error of mean). Comparison of means was obtained from Tukey’s honest significant difference test. Means with the same letter are not significantly different at (*P* < 0.05).

**Figure 7:**
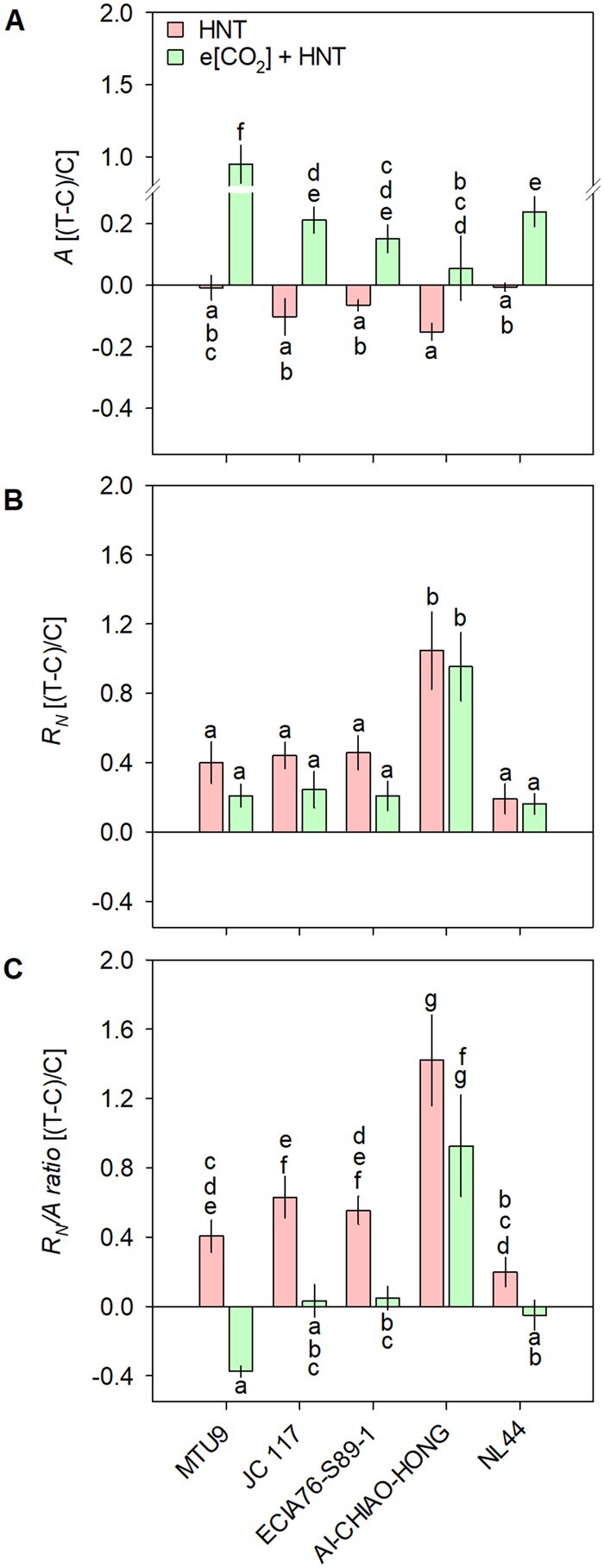
Phenotypic variation in gas exchange traits under HNT and e[CO_2_] + HNT. Relative change in photosynthesis (*A*; A), night respiration (*R_N_*; B), and *R_N_/A* ratio (C) in rice genotypes MTU9, JC 117, ECIA76-S89-1 (HCR), AI-CHIAO-HONG (LCR) and NL44 (HST) under HNT (red columns) and e[CO_2_] + HNT combination (green columns) compared to their respective controls (ambient temperature and ambient [CO_2_]). Each vertical column represents mean of five replicates. Bars indicate ±se. Comparison of means was obtained from Tukey’s honest significant difference test. Means with the same letter are not significantly different at (*P* <  0.05). (Control (C) and treatment (T)).

HNT significantly reduced grain yield and yield components across the genotypes under semi-arid conditions (Table 2), which is in line with previous findings from sub-tropical conditions in the Philippines (Shi et al., 2013, 2017; Bahuguna et al., 2017). Augmented respiratory carbon loss and reduced activity of sink (grain) enzymes are indicated as major reasons leading to lower biomass and yield under HNT (Bahuguna et al., 2017; Shi et al., 2017; Impa et al., 2019). Interestingly, phenotypic responses varied significantly under e[CO_2_] + HNT treatment, wherein HCR genotypes overcame HNT-induced losses but LCR genotype did not show any improvement (Figure 6 and Table 2). A systematic approach of screening [CO_2_] responsiveness across LPD and validating under e[CO_2_] conditions helped discover promising [CO_2_] responsive genotypes that could maintain significantly higher photosynthesis, thus leading to increased carbon fixation with adequate substrate ([CO_2_]) supply (Figure 7, A–C; Supplemental Figure S9) and maintained carbon balance (Figure 7, A–C; Supplemental Figure S10, A and B). Moreover, higher plasticity in sink size (panicle number and panicle weight) in HCR genotypes would allow for more carbon to be fixed through increased photosynthesis (Figure 7, A–C; Table 2). Hence, it can be reasonably hypothesized that additional carbon fixed could compensate the expected carbon loss through respiration (Figure 7, A–C; Supplemental Figure S10B), highlighting the ability of [CO_2_]-responsive cultivars to maintain higher biomass and yield even under HNT (Table 2).

Under e[CO_2_] + HNT condition, e[CO_2_] was the major driver influencing phenotypic response of HCR genotypes, while LCR genotype was severely affected by HNT despite exposure to e[CO_2_]. Previous studies on e[CO_2_] + HNT interaction, using one rice genotype under controlled environment, reported that HNT negated stimulatory effect of e[CO_2_] on growth and yield (Cheng et al., 2009, 2010), in line with our observation related to LCR genotype. Conversely, HCR genotypes outperformed HST genotype NL44 (Figure 7, A–C). Considering that reduction in [CO_2_] concentration in the atmosphere is an unlikely event in current and future climate, active selection, and breeding for [CO_2_] responsiveness is a promising route to achieve higher yield in C_3_ crops under warmer nights. However, phenotypic plasticity in response to other resources such as light, nutrients, etc., should be carefully observed to determine the relevance of this approach in crop improvement programs, as a decline in performance under resource poor conditions cannot be ruled out.

### Breeding for higher [CO_2_] responsiveness to overcome HNT-induced loss in yield

Despite unprecedented rise in atmospheric [CO_2_] from post-industrial period, breeding efforts for yield improvement have not considered maximizing [CO_2_] responsiveness in C_3_ crops. Lack of systematic methodology for phenotyping genetically diverse germplasm for [CO_2_] responsiveness and limited understanding of traits associated with [CO_2_] response has hampered the opportunity to benefit from rising [CO_2_] on growth and yield of C_3_ crops (Ziska et al., 2012). Simple, inexpensive, and efficient screening of large and diverse populations using LPD has been tested in different C_3_ crops. However, our findings indicate that using CRI involving different growth, yield, and physiological traits will help in identifying the most promising HCR donors by eliminating potential tradeoffs. Developing breeding populations using such HCR donors identified through composite response would increase the ability to map genomic regions responsive to [CO_2_] that can be utilized to develop varieties to benefit from current and future [CO_2_] rich environments. Rice varieties developed with significantly higher [CO_2_] responsiveness can help in increased carbon fixation and can compensate for HNT-induced losses in biomass and grain yield. Thus, HCR cultivars avoiding the HNT effect may be considered as an alternative strategy from tolerance to HNT.

## Concluding remarks

Planting geometry as an effective and surrogate method to screen [CO_2_] responsive genotypes was confirmed and further improved by integrating phenotypic plasticity from different traits to overcome potential tradeoffs associated with conclusions drawn based on individual traits. Findings from contrasting genetic backgrounds related to [CO_2_] responsiveness revealed that e[CO_2_] could ameliorate HNT induced losses in HCR genotypes, while LCR genotypes did not respond to e[CO_2_], resulting in significant loss of yield and biomass. Investigating molecular mechanism(s) regulating plant responses to HNT across HCR and LCR genotypes would provide insights into pathways that help maintain positive carbon balance under HNT environments. Our findings provide a solution to overcome sensitivity of rice to HNT by active selection of genotypes and developing varieties that are highly responsive to [CO_2_]. Screening of C_3_ crops for [CO_2_] responsiveness, mapping genomic regions, and developing breeder-friendly markers from populations developed using HCR donors are a promising route to protect rice and other C_3_ crops from HNT-induced yield losses, under current and future warmer climates.

## Materials and methods

### Site description and experimental details

The study was conducted at the experimental farm of ICAR-Indian Agricultural Research Institute, New Delhi located at 28°35′N latitude, 77°12′E longitude and at an altitude of 228.16 m above mean sea level. The climate of the experimental site is characterized as semi-arid with dry hot summer and mild winters. The crop season (Kharif) for rice (*O.* *sativa* L.) cultivation is from July to October. Three independent experiments were performed during the Kharif seasons of 2016, 2017, and 2018. A rice diversity panel consisting of 194 *O. sativa* indica accessions (Supplemental Table S4) was used for the study. This panel was assembled at the International Rice Research Institute, Philippines.

### Experiment I

#### Screening rice diversity panel for [CO_2_] responsiveness under LPD

During 2016, rice nursery was raised in the field following the dry-bed method. The nursery was irrigated frequently to maintain seedlings under well-watered nonstress conditions. Upper layer of the soil was mixed with vermicompost (0.9 kg m^−2^) and N, P, and K (urea [1 g m^−2^], diammonium phosphate [2.5 g m^−2^], and muriate of potash [1 g m^−2^], respectively) were applied to ensure adequate nutrients supply. Twenty-one-day-old seedlings were transplanted on July 20, 2016, with two seedlings hill^−1^ at two planting densities viz. NPD (20 cm between rows × 20 cm between hills i.e. 25 plants m^−2^), and LPD (40 cm between rows × 20 cm between plants i.e. 12.5 plants m^−2^) as described previously (Shimono, 2011; Shimono et al., 2014). The experiment was arranged in a randomized complete block design with three replicates each for NPD and LPD, resulting in a total of 1,164 plots. Each genotype within each replicate consisted of 18 plants arranged in three rows, each accommodating six hills. Fully flooded conditions were maintained throughout the growing season, across both the treatments. Urea (N), single superphosphate (P), and muriate of potash (K) were applied at the rate of 120, 40, and 60 kg ha^−1^, respectively. The entire dose of P and K fertilizers was applied as basal dose while N fertilizer was applied in three splits, i.e. 50% before transplanting, 25% at active tillering, and the remaining 25% at the heading stage (Bahuguna et al., 2015). No major insect and pest infestation were observed during the experiment. Non-destructive physiological observations such as greenness index (SPAD value), gas exchange measurements were taken on 191 genotypes when respective genotypes reached 50% flowering stage. At physiological maturity, five plants from the middle row were harvested from each genotype in each replicate.

### Experiment II

#### [CO_2_] responsiveness under FACE rings

In 2017, a contrasting set of 23 rice genotypes was selected based on their varying responsiveness (phenotypic plasticity) to planting geometry from Experiment I. Nursery was sown following the dry-bed method and maintained with adequate moisture and nutrients as described in Experiment I. Twenty-one-day-old seedlings were transplanted on July 21, 2017 in three field-based FACE rings with each ring having an area of 144 m^2^ (with actual area of 80 m^2^ within FACE ring and the rest planted with border plants), and each ring was considered as an independent replicate (Supplemental Figures S12, A and S13). Row to row and plant to plant distance was maintained at 20 cm × 20 cm in the FACE rings. The same set of 23 genotypes was transplanted on the same date at two planting densities viz. NPD and LPD (in open field conditions) with plant to plant and row to row distance maintained as in Experiment I for realistic comparison between FACE and LPD response within the same cropping season. All the agronomic practices and fertilizer applications for NPD, LPD, and FACE rings were same as in Experiment I. All three treatments (NPD, LPD, and FACE) were arranged in a randomized block design with three replicates for each treatment. Each genotype was grown in 5 m^2^ (1 m × 5 m) area (∼125 hills) in each replicate.

Each octagonal-shaped FACE ring was equipped with eight 4-m long plenums made up of flexible poly vinyl chloride pipe of 20 cm diameter, with independent control of [CO_2_] release every day from 0600 to 1800 h. Details of functioning of FACE rings are provided in Supplemental Figure S12. Elevated [CO_2_] treatment (∼300 µmol mol^−1^ higher than ambient) was started seven days after transplanting and continued until physiological maturity. Non-destructive physiological observations including SPAD value and gas exchange traits were recorded from all the 23 rice genotypes from FACE, LPD, and NPD treatments at 50% flowering stage. At physiological maturity, five plants from the middle row of each replicate for each genotype and treatment were harvested for analyzing grain yield and related parameters.

### Experiment III

#### Assessment of rice response to e[CO_2_] + HNT interaction

During 2018, a contrasting set of five genotypes, screened and validated under Experiments I and II, respectively, was tested for HNT and e[CO_2_] + HNT interaction. Three HCR, one LCR, and one known heat stress tolerant (HST) cultivar Nerica L44 (Bahuguna et al., 2015; Chaturvedi et al., 2017) were included. The aim was to quantify their response to HNT and e[CO_2_] + HNT interaction using field-based HNT tents facility at IARI, New Delhi (Supplemental Figure S13). Briefly, each custom-designed HNT tent (8 m [length] × 4 m [width] × 3 m [height]) was made up of GI pipes fitted on grounded concrete base at 1m depth below surface soil. In each tent, an oil-filled heat radiator (GHROFAFK290, Havells, India) was placed in the middle and fixed on wooden platform which was 0.5 m above the ground. These heat radiators in each tent were turned on daily at 1800 h to raise air temperature within HNT tents. Tents were covered manually at 1800 h with extreme duty waterproof, puncture-resistant vinyl tarpaulin (50 mils thickness) to trap the heat from radiators within the tent. The standing water provided a leak-proof seal at the bottom of the tent and was highly effective in retaining heat generated by the radiators, similar to Shi et al. (2013). There were two exhaust vent fans (2,200 rpm) at each end of the tents operating throughout the night for even circulation of heated air inside the HNT tent. Furthermore, the tents were opened manually at 0600 h, heaters and exhaust vents were turned off, and the tarpaulin was rolled up to expose the plants to natural ambient conditions during the daytime (Supplemental Figure S13).

For e[CO_2_] + HNT treatment, a unit of HNT tent was connected to the same [CO_2_] release system used for FACE rings in Experiment II. Six plenums (each 3-m long GI-pipes equipped with pressure nozzles) were connected to the HNT tent through a separate set of solenoid valves, flow meters, and [CO_2_] regulators to control [CO_2_] release into the tent. Each tent was equipped with temperature and RH sensors (Ambitronics, India, Mumbai, Ltd.) and [CO_2_] monitoring system (Vaisala GMP 343 sensor with data logger). Air temperature, RH, and e[CO_2_] were measured at 30 min intervals. Temperature and RH sensors were housed in Micrometeorological Instrument for the Near-Canopy Environment of Rice obtained from National Institute for Agro-Environmental Sciences, Tsukuba, Japan (Fukuoka et al., 2012), and fixed on a tripod stand. Vaisala GMP343 [CO_2_] probe was fixed at the center of the tent to measure the [CO_2_] level at the canopy height (Supplemental Figures S12B and S13).

Seedlings of five rice genotypes were raised in field nursery similar to Experiments I and II and 21-one-day-old seedlings were transplanted with two seedlings hill^−1^ in control, HNT, and e[CO_2_] + HNT tents on July 20, 2018. Each HNT tent having an area of 32 m^2^ (8 m × 4 m) accommodated five genotypes planted following a randomized design, with each genotype occupying 1.5 m^2^ (1 m × 1.5 m), replicated three times within the tent (Supplemental Figure S12B). Plant to plant and row to row spacing was fixed at 20 cm. Elevated [CO_2_] treatment was initiated from the early vegetative stage (7 d after transplanting) and continued until physiological maturity, while HNT treatment was initiated at the panicle initiation and continued until physiological maturity following Shi et al. (2013) and Bahuguna et al. (2017). Gas exchange measurements were recorded during active grain-filling period (5 d after 100% flowering). At physiological maturity, eight plants from the middle rows were harvested from each replication, treatment, and genotype for recording yield and yield-related parameters.

### Observations

#### Greenness index (SPAD)

Greenness index (SPAD value) was measured using a self-calibrating SPAD-502 chlorophyll meter (Konica Minolta Sensing Inc., Tokyo, Japan) on flag leaf at 50% flowering between 0900 and 1130 h, in all experiments. Three replicate readings were taken on one side of the midrib of the flag leaf and averaged for a single observation (Yang et al., 2014).

#### Gas exchange measurements

Gas exchange measurements were obtained from the flag leaf of three randomly selected plants using portable photosynthesis system LI-6400XT (LI-COR Inc., Lincoln, NE, USA) between 0900 to 1130 h on bright sunny days. A day before the start of flowering, genotypes were identified and tagged for gas exchange measurements. [CO_2_] concentration of the sample chamber was adjusted to 400 µmol mol^−1^ using a [CO_2_] mixer with the LI-COR [CO_2_] injection system. A constant flow rate of 400 μmol s^−1^ and a near-saturating photosynthetic photon flux density of 1,200 µmol m^−2^ s^−1^ from an inbuilt LI-6400XT LED light source was maintained. During all the measurements, humidity in the sample chamber was controlled through a desiccant and maintained close to 65%. Conversely, night-time respiration was measured in Experiment III between 2300 and 0100 h, as described in Bahuguna et al. (2017), on the same leaf used for day-time gas exchange measurement. All the parameters were kept similar to day-time measurements except the flow rate was maintained at 300 μmol s^−1^ and light source was switched off. Gas-exchange measurements, photosynthetic rate (*A*), stomatal conductance (*g_s_*), transpiration rate (*E*), and night respiration (*R_N_*) were logged after readings reached stability. Gas exchange measurements were performed at 50% flowering in Experiment I and II, while at active grain-filling stage (5 d after 100% flowering) in Experiment III.

#### Yield and yield components

Plant samples were harvested at physiological maturity in all three experiments. Five hills from Experiments I and II and eight hills from Experiment III were harvested from the middle rows of each replication and genotype to avoid any confounding border effects. Number of tillers and panicles were counted manually from each hill. Panicles were separated from all the harvested samples and only straw was oven dried at 70°C until constant weight was obtained. The panicles were sundried in net-bags and weighed using analytical balance (model: BSA124S-CW, Sartorius AG, Germany). Grain yield was determined for each hill and adjusted to the standard moisture content (0.14g H_2_O g^−1^) (Bahuguna et al., 2017). The above-ground total biomass was the combined dry matter of straw and panicles. TGW was calculated by weighing three replicate samples of 1,000 seeds each taken randomly for each genotype and treatment. HI was calculated as the ratio of the yield to the total aboveground biomass including grain yield (Sinclair, 1998).

### Response index (RI)

Phenotypic plasticity in the rice genotypes under LPD and e[CO_2_] was estimated as a RI modified from Blum et al. (1989) to differentiate genotypes based on different individual phenotypic traits such as tillers per hill, panicles per hill, grain yield, total biomass, HI, and photosynthesis (*A*).

RI for an individual trait was calculated as

$$\text{RI}=\left(1-Y_{T}/Y_{C}\right)/\left(1-X_{T}/X_{C}\right)$$

where *Y*_T_ is trait value of a genotype under LPD or e[CO_2_], *Y*_C_ is trait value of the same genotype under NPD, *X*_T_ is the average trait value from all the genotypes under LPD or e[CO_2_], and *X*_C_ is the average trait value of all the genotypes under NPD.

CRI was calculated as

$$\text{CRI}=\left(\left[\left({\text{RI}\;\text{trait}}_{1}\right)+\left({\text{RI}\;\text{trait}}_{2}\right)+\left({\text{RI}\;\text{trait}}_{3}\right)\;\ldots \ldots .+\left({\text{RI}\;\text{trait}}_{n}\right)\right]/n\right)$$

### Statistical analysis

Two-factor analysis of variance was employed to determine genotype (G), treatment (T), and their interaction (GxT), and comparison of means was tested (*P* < 0.05) by Tukey’s post hoc test using GenStat release version 12.1 (Rothamsted Experimental Station, Harpenden, UK). A multivariate principal component analysis was performed in R software (R Studio version 1.3.1056) and biplots were prepared from the PCs results using “factoextra” package for yield and physiological traits from different rice genotypes across the treatments.

## Supplemental data

The following materials are available in the online version of this article.

**Supplemental Figure S1.** Meteorological data during 2016, 2017, and 2018.

**Supplemental Figure S2.** Phenotypic variation of greenness index (SPAD value) under different planting densities and e[CO_2_].

**Supplemental Figure S3.** Phenotypic variation in gas exchange traits under normal and LPD.

**Supplemental Figure S4.** Summary of phenotypic variation in gas exchange traits under normal and LPD.

**Supplemental Figure S5.** Phenotypic variation in growth and yield component traits measured as RI.

**Supplemental Figure S6.** CRI calculated for 191 rice genotypes under LPD.

**Supplemental Figure S7.** Principal component analysis of grain yield, yield components, greenness index, and gas exchange traits.

**Supplemental Figure S8.** Relationship between the response of 23 rice genotypes to LPD and e[CO_2_].

**Supplemental Figure S9.** Effect of HNT and e[CO_2_] + HNT interaction on gas exchange traits.

**Supplemental Figure S10.** Effect of HNT and e[CO_2_] + HNT interaction on night respiration and night respiration/photosynthesis ratio.

**Supplemental Figure S11.** Linear regression analysis of photosynthesis under LPD and e[CO_2_].

**Supplemental Figure S12.** Schematic diagram showing layout of one of the three FACE rings and HNT tents.

**Supplemental Figure S13.** HNT tents and FACE facility.

**Supplemental Table S1**. Descriptive statistics and the significance (Fischer’s test summary) for different yield and physiological traits under NPD and LPD.

**Supplemental Table S2**. Grain yield and yield components of 23 rice genotypes phenotyped under NPD, LPD and e[CO2].

**Supplemental Table S3**. ANOVA for leaf photosynthesis, stomatal conductance and transpiration for experiment III in 2018.

**Supplemental Table S4**. List of rice accessions in the Oryza sativa subsp. indica diversity panel assembled at IRRI.

## Supplementary Material

kiab470_Supplementary_Data

## Notes

The author responsible for distribution of materials integral to the findings presented in this article in accordance with the policy described in the Instructions for Authors. (https://academic.oup.com/plphys/pages/general-instructions) is: S.V. Krishna Jagadish, kjagadish@ksu.edu.
