# Supplementary material for: Carbon dioxide responsiveness mitigates rice yield loss under high night temperature
Source: Plant Physiol. 2021 Oct 13;188(1):285–300. doi: 10.1093/plphys/kiab470 (PMC8774858; doi:10.1093/plphys/kiab470)
Supplement: kiab470_Supplementary_Data [file kiab470_supplementary_data.docx]

**Supplemental Data**

**Title**: Carbon-dioxide responsiveness mitigates rice yield loss under high night temperature

**Authors**: Rajeev N. Bahuguna, Ashish K. Chaturvedi, Madan Pal, Viswanathan Chinnusamy, S.V. Krishna Jagadish, Ashwani Pareek

**

**

**Supplemental Figure S1.** Meteorological data during 2016, 2017 and 2018. Daily mean day and night temperature, daily mean relative humidity and rainfall pattern recorded during the *kharif* season (June to October) in 2016 (a), 2017 (b) and 2018 (c). All the parameters were recorded from sowing till physiological maturity.

**Supplemental Figure S2**. Phenotypic variation of greenness index (SPAD value) under different planting densities and elevated [CO_2_]. Box-plot showing the phenotypic variation of greenness index (SPAD value) in 191 rice genotypes under normal planting density (NPD) and low planting density (LPD) in 2016 (a) and variation of greenness index in 23 rice genotypes under NPD, LPD and e[CO_2_] in 2017 (b). Inside the boxplot, the bold and dotted lines represent the median and the mean of the population, respectively. Box edges represent upper and lower quantiles, and whiskers are 1.5× the quantile of the data. Outliers are indicated in open circles. Levels of significance for genotype (G), treatment (T), and their interaction (GxT) effects from *ANOVA* are given with LSD (least significant difference [P<0.05]) (***, P<0.001), ns- non-significant.

**Supplemental Figure S3**. Phenotypic variation in gas exchange traits under normal and low planting density. Box-plot showing phenotypic variation of leaf photosynthetic rate (*A,* a), stomatal conductance (*g_s_*, b), and transpiration rate (*E*, c) in 191 rice genotypes under normal planting density (NPD) and low planting density (LPD) during 2016. Inside boxplot, the solid and dashed lines represent the median and the mean of the population, respectively. Box edges represent upper and lower quantiles, and whiskers are 1.5× the quantile of the data. Outliers are shown as open circles. Levels of significance for genotype (G) and treatment (T) effects from *ANOVA* are given with LSD (least significant difference) value (P<0.05).[Significance; ***, P<0.001]

**
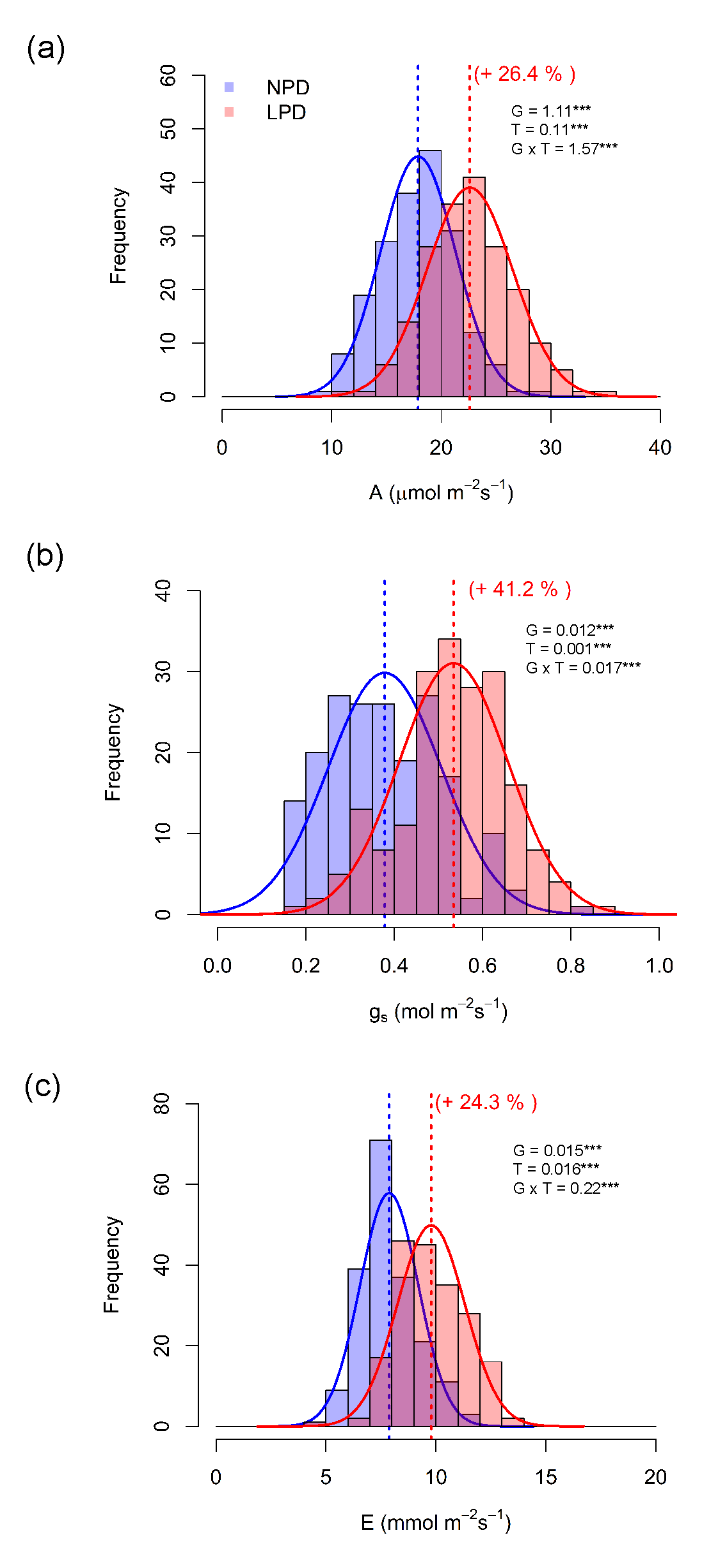
**

**Supplemental Figure S4**. Summary of phenotypic variation in gas exchange traits under normal and low planting density. Overlying histograms with normal distribution curves (normal planting density: NPD, blue line, bars; low planting density: LPD, orange line, bars and overlap between treatments with the lower frequency value, pink bars) showing the phenotypic distribution of gas exchange traits - leaf photosynthetic rate (*A,* a), stomatal conductance (*g_s_*, b) and transpiration rate (*E*, c) in 191 rice genotypes during 2016. The vertical lines in the histograms show population mean values in NPD (blue) and LPD (orange) conditions, and values in parentheses represent the significant percentage change (+, increase) in LPD as compared to NPD. Levels of significance for genotype (G), treatment (T), and their interaction (GxT) effects from ANOVA are given with LSD (least significant difference [P<0.05]) value in the histograms (***, P<0.001).





**Supplemental Figure S5. Phenotypic variation in growth and yield component traits measured as response index.** Response index (RI) calculated for plant height (a), tillers per hill (b), panicles per hill (c), grain yield per hill (d) and total biomass per hill (e) in 194 rice genotypes under low planting density (LPD). Vertical lines show genotypes arranged from least responsive to most responsive for each trait. Response index was calculated as per Blum et al. (1989).


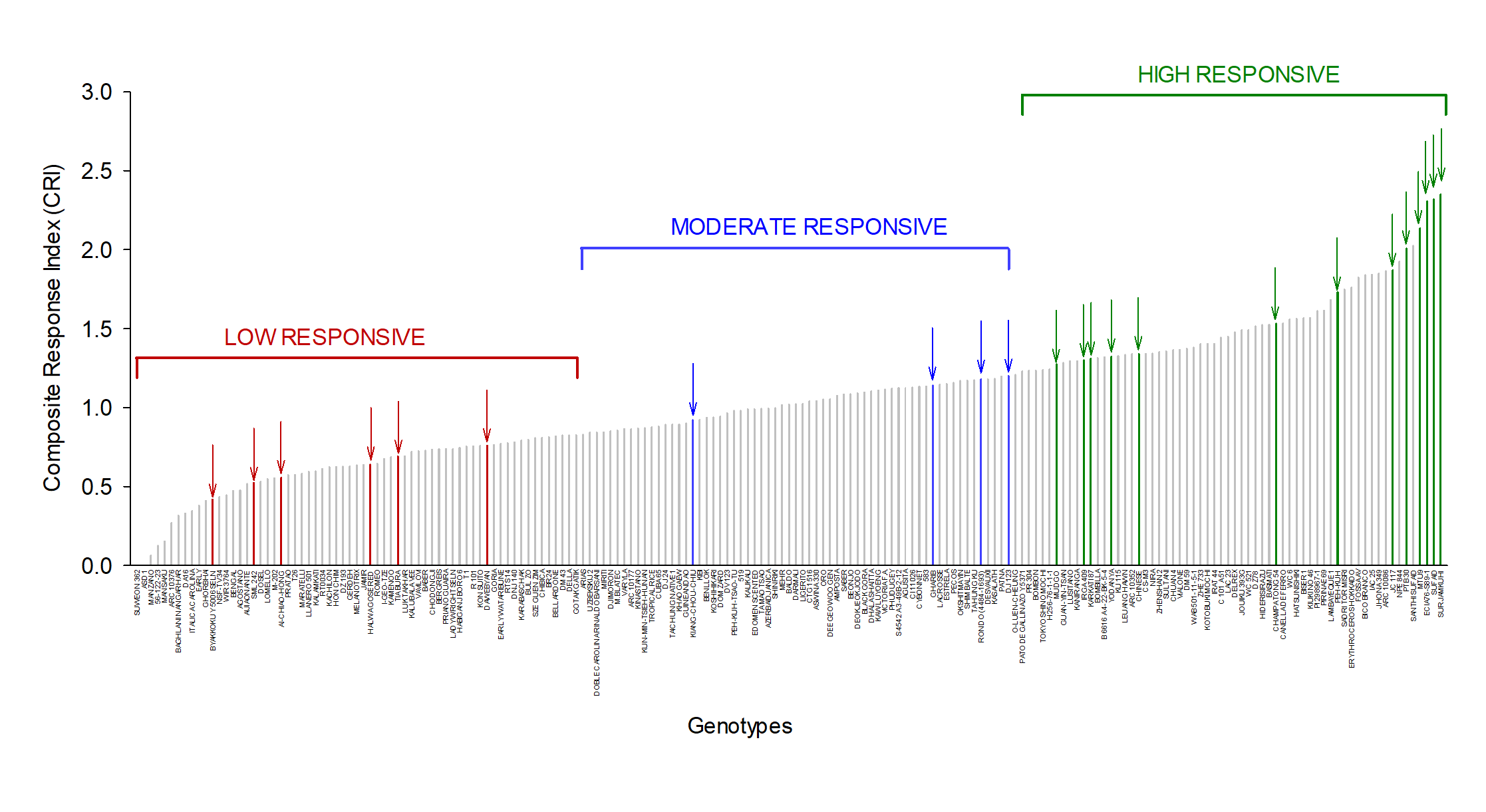


**Supplemental Figure S6.** Composite response index (CRI) calculated for 191 rice genotypes under low planting density (LPD). Response index was individually calculated for yield components (tillers per hill, panicle per hill, grain yield per hill, total biomass per hill, harvest index) and flag leaf photosynthesis (*A*) and averaged to obtain CRI for each genotype. Vertical lines show genotypes arranged from low responsive to high responsive. All genotypes were divided in to three groups as high, moderate and low responsive based on CRI value. Genotypes were selected from each group based on CRI and with similar phenology (days to heading) for experiment II. Genotypes selected for further validation under elevated [CO_2_] are indicated with inverted vertical arrows in high responsive (green), moderate responsive (blue) and low responsive (red) groups, respectively.


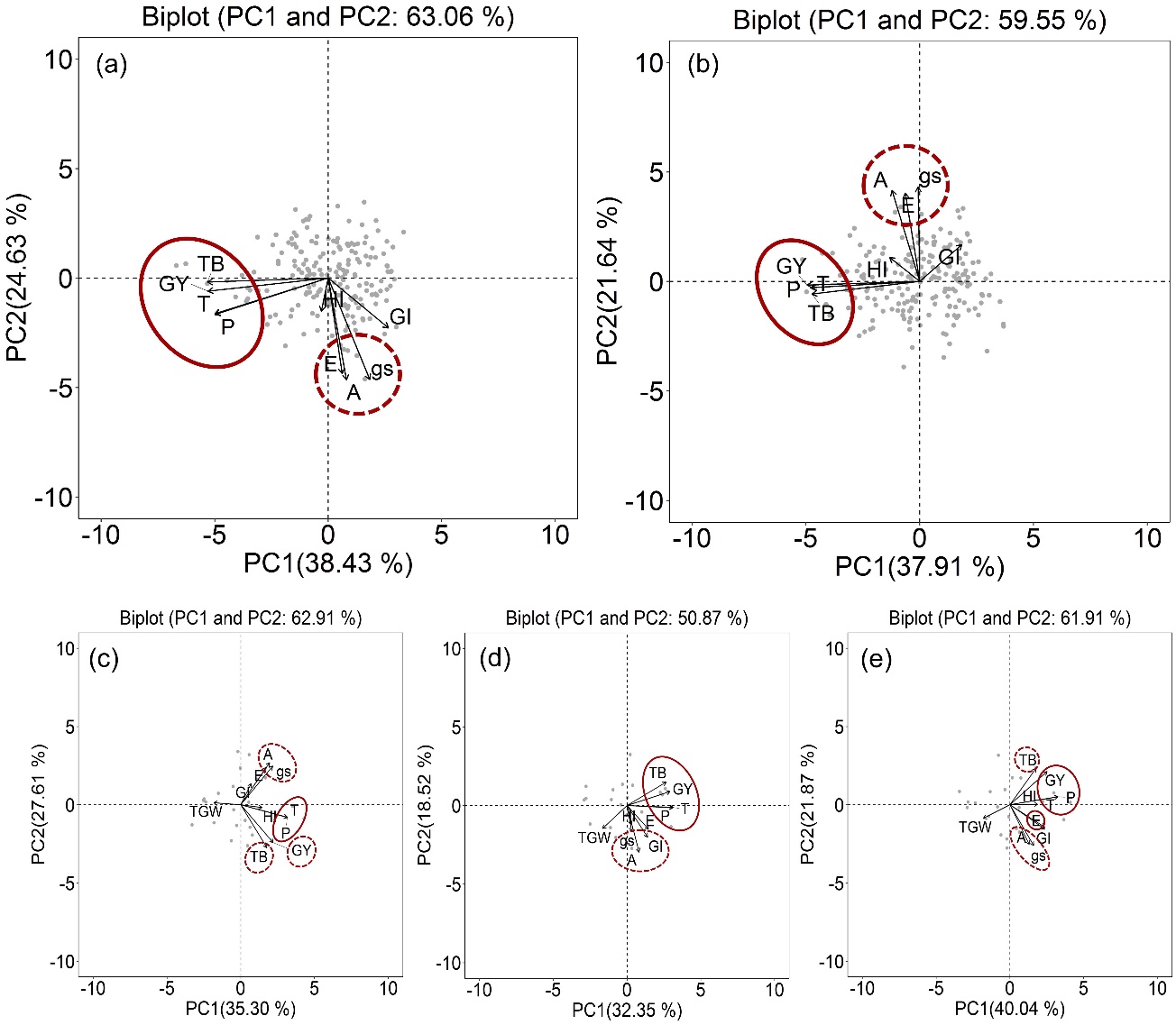


**Supplemental Figure S7.** Principal component analysis of grain yield, yield components, greenness index and gas exchange traits (n=191) during 2016 (a, b); grain yield, yield components, greenness index and gas exchange traits (n=23) during 2017 (c, d, e) with first two principal components (PC1 and PC2) in normal planting density (NPD) (a, c), low planting density (LPD) (b, d) and elevated CO_2_ (e[CO_2_]) (e). The traits marked inside the solid circle/ellipses contributed more to the variation explained by PC1 and those marked inside the dashed ellipses to PC2. ‘n’ represents number of genotypes. T, tillers hill^-1^; P, panicles hill^-1^; GY, grain yield hill^-1^; TB, total biomass hill^-1^; TGW, thousand grain weight; HI, harvest index; GI, greenness index (SPAD value); *A*, leaf photosynthetic rate; *g_s_*, leaf stomatal conductance; and *E*, leaf transpiration rate

**
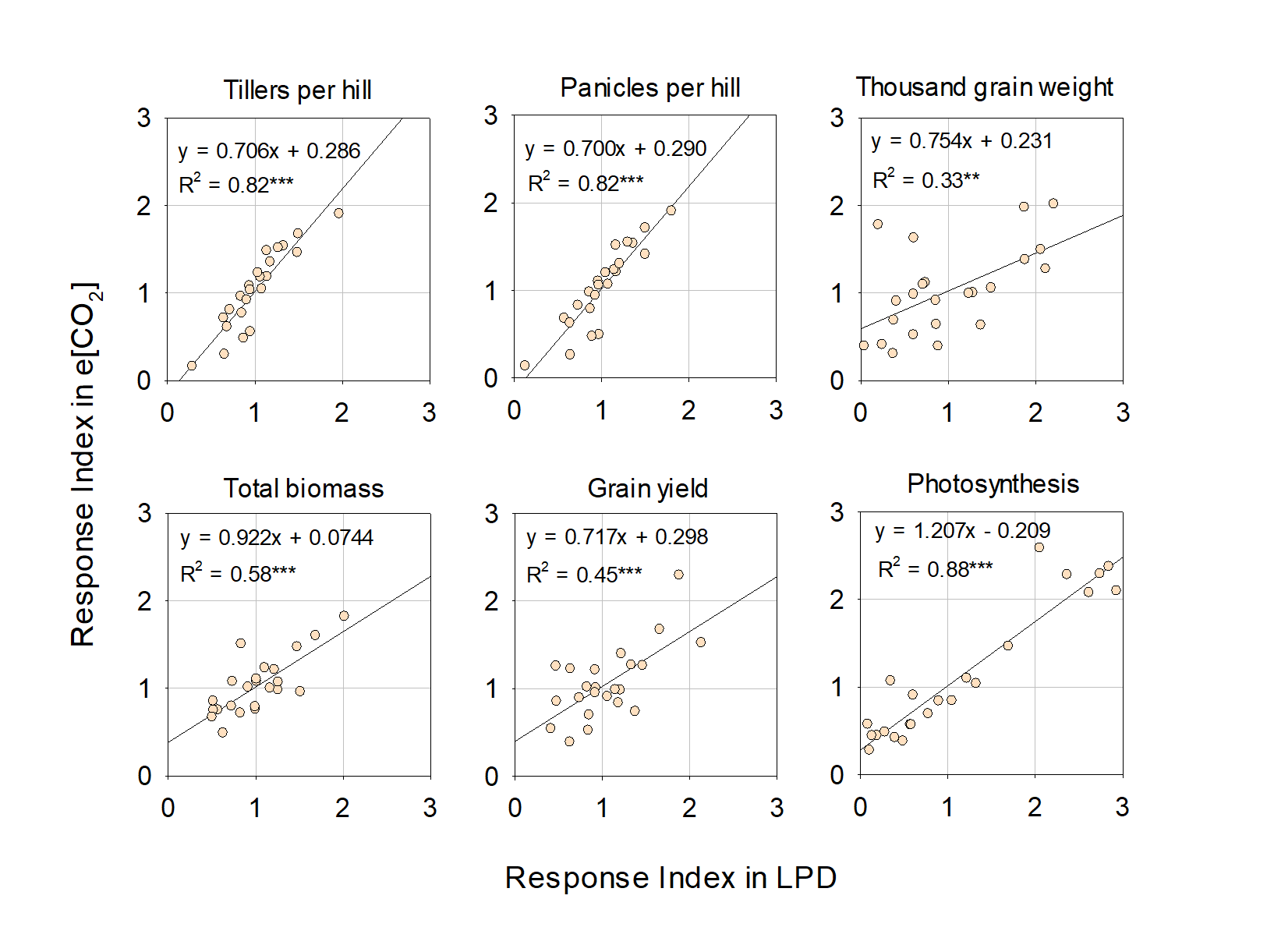
**

**Supplemental Figure** **S8.** Relationship between response of 23 rice genotypes to low planting density (LPD) and elevated [CO_2_] during 2017. Responsiveness of grain yield, yield components (tillers hill^-1^, panicles hill^-1^, grain yield hill^-1^, total biomass hill^-1^, thousand grain weight) and photosynthesis were calculated individually for LPD and e[CO_2_]. Each circle represents a genotype. [Significance for linear regression analysis ***, P<0.001; **, P<0.01]

**
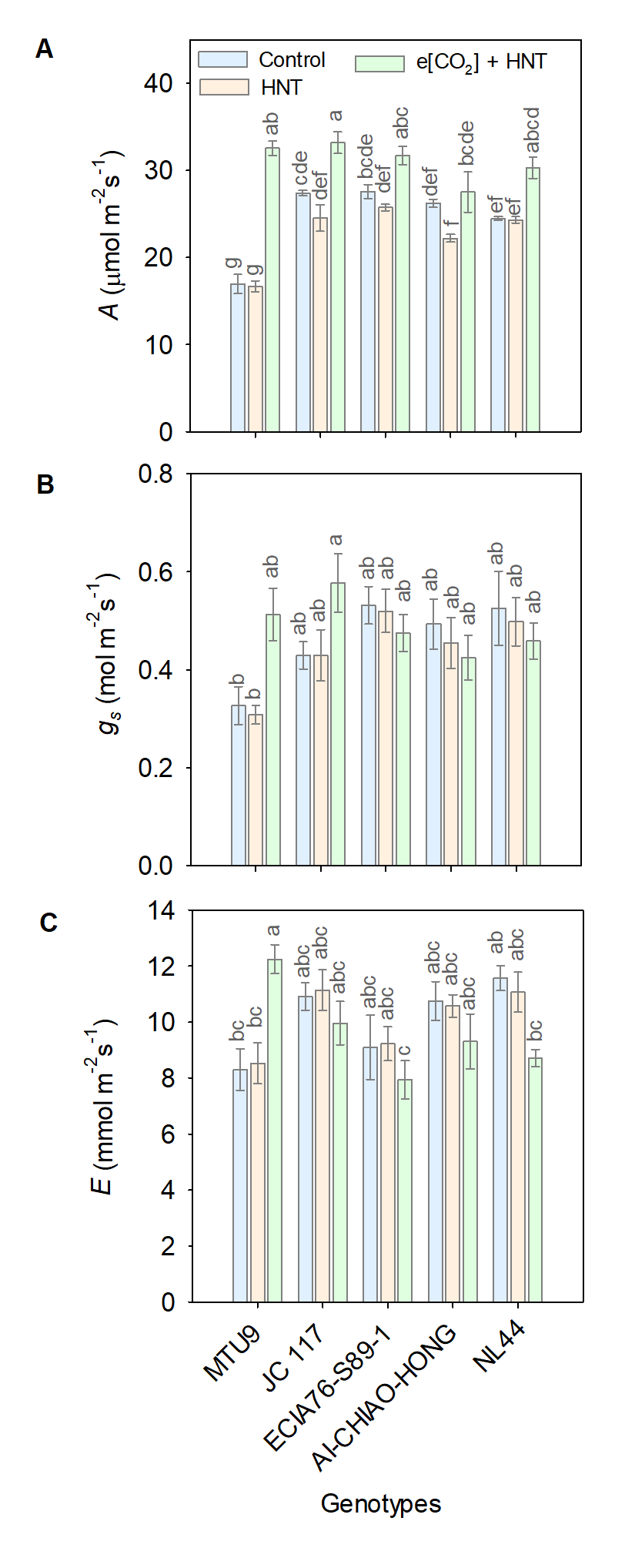
**

**Supplemental Figure S9**. Effect of high night temperature (HNT) and e[CO_2_] + HNT interaction on gas exchange traits. Leaf photosynthetic rate (*A,* a), stomatal conductance (*g_s_*, b) and transpiration rate (*E*, c) of rice genotypes MTU9, JC117, ECIA76-S89-1 (high responsive to e[CO_2_], HCR), AI-CHIO-HONG (least responsive to e[CO_2_], LCR) and NL-44 (heat stress tolerant, HST) under HNT and e[CO_2_] + HNT combination compared to their respective controls (ambient temperature and ambient [CO_2_]) during 2018. Data shown are mean of five replicates ± SE. Comparison of means was obtained from Tukey’s honest significant difference test. Means with the same letter are not significantly different at (P<0.05).

**
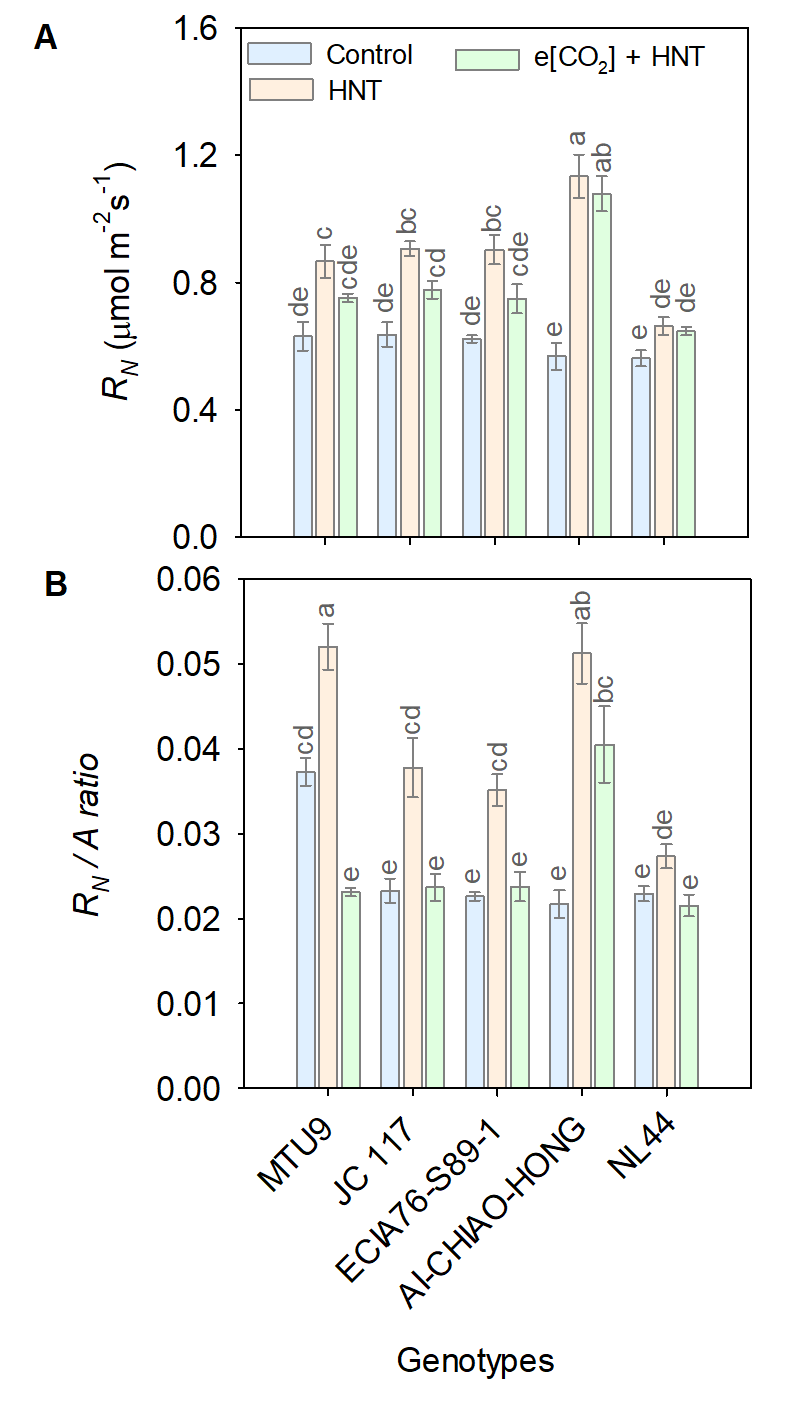
**

**Supplemental Figure S10**. Effect of high night temperature (HNT) and e[CO_2_] + HNT interaction on night respiration and night respiration/photosynthesis ratio. Leaf night respiration (*R_N_*, a) and *R_N_/A* ratio (b) of rice genotypes MTU9, JC117, ECIA76-S89-1 (high responsive to e[CO_2_], HCR), AI-CHIO-HONG (least responsive to e[CO_2_], LCR) and NL-44 (heat stress tolerant, HST) under HNT and e[CO_2_] + HNT combination as compared to their respective controls (ambient temperature and ambient [CO_2_]) during 2018. Data shown are mean of five replicates ± SE. Comparison of means was obtained from Tukey’s honest significant difference test. Means with the same letter are not significantly different at (P<0.05).

**
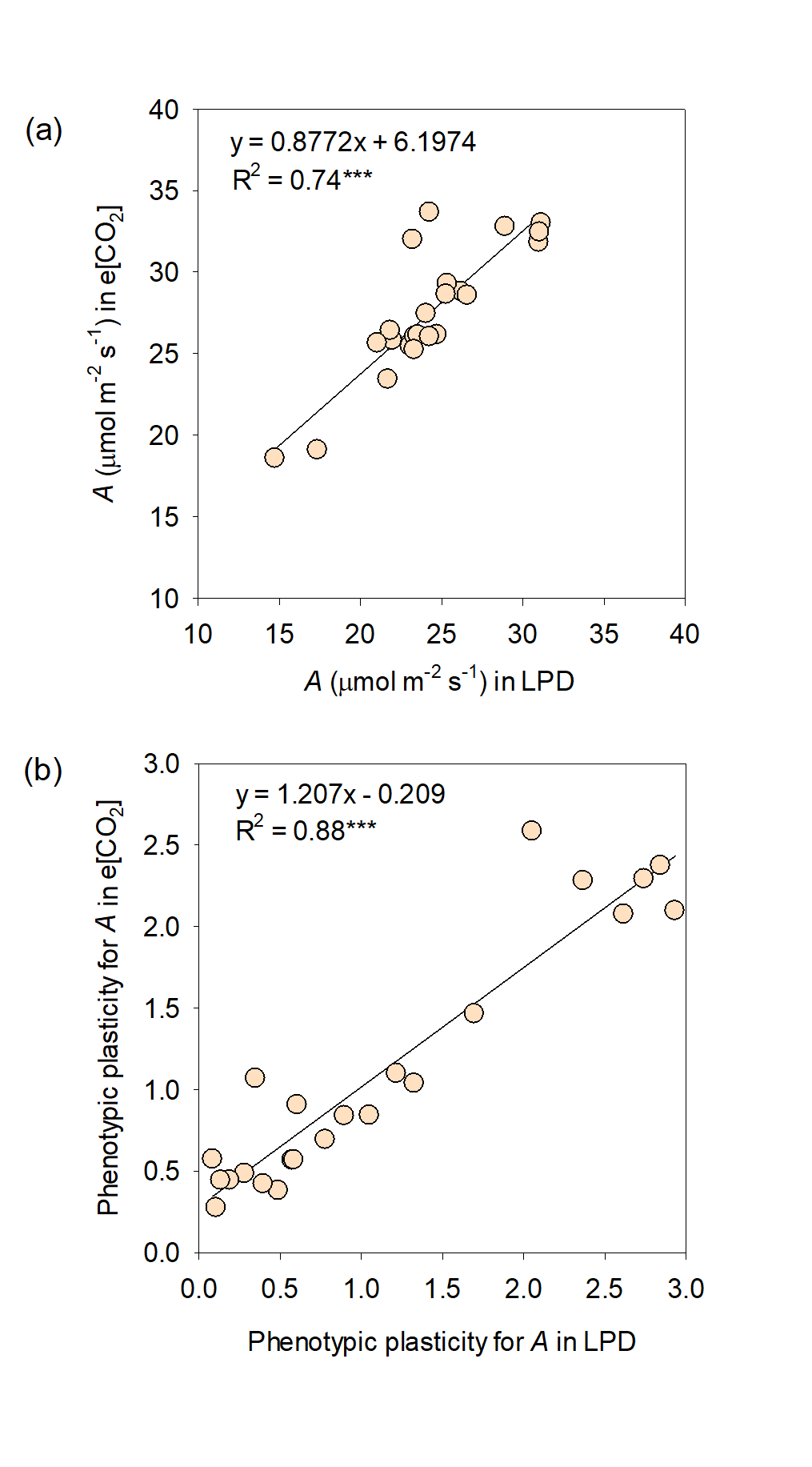
**

**Supplemental Figure S11**. Linear regression analysis of photosynthesis under low planting density (LPD) and elevated [CO_2_] (e[CO_2_]). Relationship between photosynthesis (*A*, a) and phenotypic plasticity for *A* (b) of 23 rice genotypes to LPD and e[CO_2_] during 2017. Each circle represents a mean value of a genotype. [Significance for linear regression analysis, ***, P<0.001]


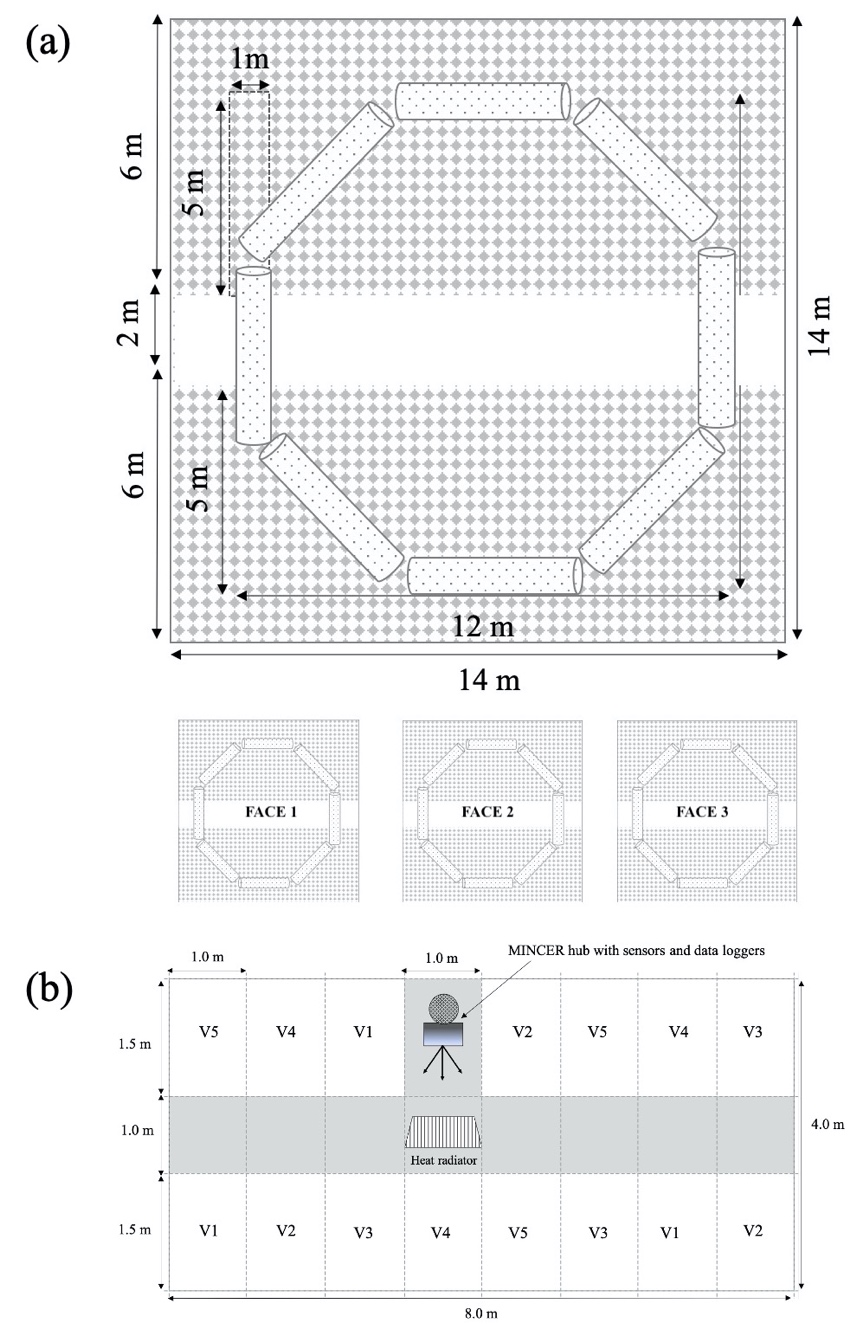


**Supplemental Figure S12.** Schematic diagram showing layout of one of the three FACE rings (a) and HNT tent (b). Each FACE ring occupied an area of 144 m^2^ (12 m x 12 m). Air compressors with 200 litres capacity were used to pump air mixed with [CO_2_] into FACE ring through Galvanised Iron (GI) pipes. Pure CO_2_ gas (99.7%, v/v [CO_2_] with less than 10 ppm CO; M/S Gas Associates, New Delhi, India) was released through solenoid valves (Fluidtecq Pneumatics, India) to each of the eight plenums from 25-gas cylinders connected through a manifold, valves, flow meters and fine [CO_2_] regulators, every day from 0600 to 1800h. Incoming [CO_2_] was dispersed into the FACE rings through perforated pipes with holes of 3 mm diameter. The fumigation of the [CO_2_] from the plenum was operated at the periphery for reaching the center of the field at 10 to 15 cm above the crop canopy to reduce [CO_2_] gradient with depth and to achieve uniform e[CO_2_] concentration across each FACE ring. The plenum height was adjusted with the help of adjustable stands by 40 cm intervals, reaching a final height of 1.2 m following the increase in canopy height. In the middle of each ring, sensors for wind speed, and a Vaisala GMP343 [CO_2_] probe with data logger was mounted to measure the [CO_2_] level at canopy height at every 30 min interval. Signals from these sensors were transmitted to the control room through a shielded cable. The control system was operated with microprocessor through in-built timer and data logger input integrated with necessary controls and display of [CO_2_] (Uprety et al., 2007; Sinha et al., 2009; Sinha et al., 2011). A set of 23 rice genotypes were transplanted with each genotype occupying an area of 5m^2^ (1m x 5m). Plant to plant and row to row spacing was fixed at 20 cm. A 2 m alley was maintained in the middle of each FACE ring, which provided access for physiological measurements. Genotypes were arranged randomly within each FACE ring following randomized block design where each FACE ring was treated as an independent replicate (block). Each HNT tent occupied an area of 32 m^2^ with each genotype occupying an area of 1.5 m^2^ (1m x 1.5m) in triplicate within each tent. Plant to plant and row to row spacing was fixed at 20 cm. A 1 m alley was maintained at the middle of each tent. A radiator to generate heat was placed at the middle of the tent, fixed on wooden platform, 0.5 m above the ground. Temperature, RH and CO_2_ sensors were housed in MINCER fixed on a tripod stand. A mini exhaust fan within MINCER head chamber was included to intake ambient air to measure air temperature, humidity and [CO_2_] levels. All sensors were connected to data loggers for real-time monitoring of environmental parameters within each tent.


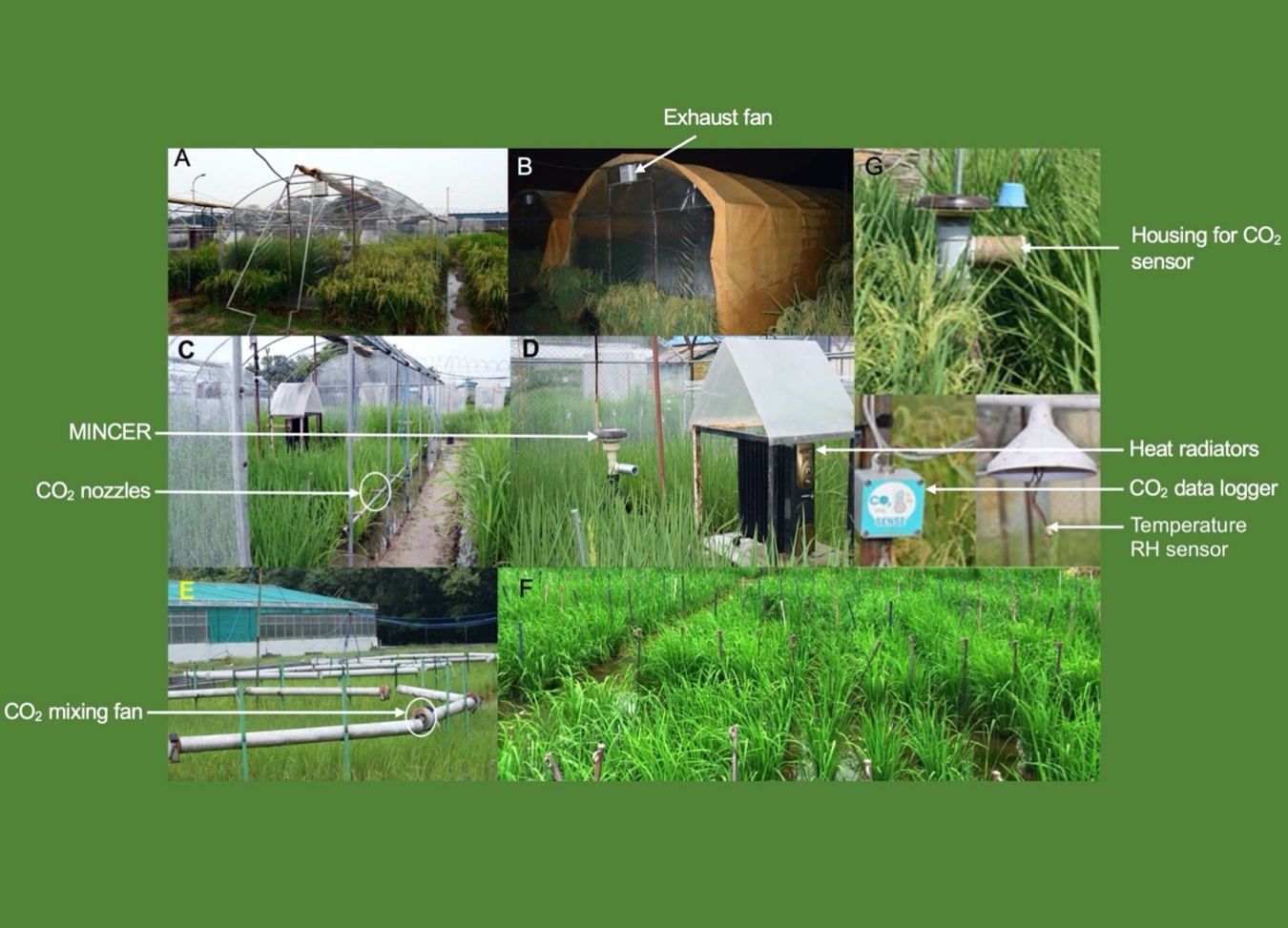


**Supplemental Figure S13.** High night temperature (HNT) tents and free air [CO_2_] enrichment (FACE) facility at Indian Agricultural Research Institute, Pusa, New Delhi, India. High night temperature (HNT) tent fully open during day time (0600 to 1800h) (A); HNT tent closed during night time (1800 to 0600h) (B); HNT tent equipped with CO_2_ enrichment facility (C), temperature and relative humidity sensors housed in MINCER and heat radiators to increase air temperature in closed tents (D), field based free air [CO_2_] enrichment (FACE) rings (E), screening of genotypes under low planting density (LPD) during 2016 (F), [CO_2_] sensor (Vaisala [CO_2_] probe GMP343) housed in MINCER for FACE and HNT tents, including [CO_2_] data logger and ambient air temperature, humidity sensors (G).

**Supplemental Table S1.** Descriptive statistics and the significance (Fischer’s test summary) for genotype (G), treatment (T) and their interactions (G × T) for different yield and physiological traits of rice diversity panel grown under normal planting density (NPD) and low planting density (LPD) during 2016. [*A*, leaf photosynthetic rate; *g_s_*, leaf stomatal conductance; *E*, leaf transpiration rate].

|  | Normal planting density | | | Low planting density | | |  | P value (Fischer’s test) | | |
| --- | --- | --- | --- | --- | --- | --- | --- | --- | --- | --- |
| Trait | Mean ± SD | Min. | Max. | Mean ± SD | Min. | Max. | % Change | G | T | G x T |
| *Growth and yield related traits* (n=194) | | | | | | | | | | |
| Plant height (cm) | 106.18±26.74 | 42.60 | 234.20 | 120.13±28.87 | 53.80 | 177.00 | 13.14 | <0.001 | <0.001 | <0.001 |
| Tillers hill^-1^ | 9.10±3.45 | 3.20 | 30.00 | 19.43±7.37 | 6.20 | 42.20 | 113.59 | <0.001 | <0.001 | <0.001 |
| Panicles hill^-1^ | 9.02±3.48 | 2.60 | 30.00 | 19.09±7.38 | 6.20 | 42.20 | 111.76 | <0.001 | <0.001 | <0.001 |
| Grain yield hill^-1^ (g) | 13.87±6.22 | 2.05 | 38.24 | 34.30±16.65 | 5.42 | 84.53 | 147.23 | <0.001 | <0.001 | <0.001 |
| Total biomass hill^-1^ (g) | 30.22±13.38 | 8.13 | 81.29 | 82.55±37.80 | 14.12 | 170.20 | 173.16 | <0.001 | <0.001 | <0.001 |
| Harvest index (%) | 0.46±0.07 | 0.20 | 0.63 | 0.42±0.08 | 0.14 | 0.61 | -9.57 | <0.001 | <0.001 | <0.001 |
| *Physiological traits* (n=191) | | | | | | | | | | |
| SPAD value | 40.19±4.87 | 21.97 | 51.20 | 43.75±5.05 | 25.43 | 52.00 | 8.86 | <0.001 | <0.001 | <0.001 |
| *A* (µmol m^-2^ s^-1^) | 17.87±3.42 | 9.89 | 28.10 | 22.60±3.93 | 11.79 | 34.64 | 26.41 | <0.001 | <0.001 | <0.001 |
| *g_s_* (mol m^-2^ s^-1^) | 0.38±0.13 | 0.16 | 0.82 | 0.53±0.12 | 0.20 | 0.87 | 41.17 | <0.001 | <0.001 | <0.001 |
| *E* (mmol m^-2^ s^-1^) | 7.87±1.33 | 4.88 | 11.44 | 9.78±1.54 | 4.88 | 13.73 | 24.25 | <0.001 | <0.001 | <0.001 |

*% C: % change (+ increase or – decrease) over control condition, n = number of genotypes* [*A*, Photosynthesis; *g_s_*, stomatal conductance; *E*, Transpiration]

**Supplemental Table S2.** Grain yield and yield components of 23 rice genotypes phenotyped under normal planting density (NPD), low planting density (LPD) and elevated [CO_2_] (e[CO_2_]) during 2017.

| **Genotype** | **Plant height (cm)** | | | **Number of tillers per hill** | | | **Number of panicles per hill** | | | **Total Biomass (g hill^-1^)** | | | **Grain yield (g hill^-1^)** | | | **1000 grain weight (g)** | | |
| --- | --- | --- | --- | --- | --- | --- | --- | --- | --- | --- | --- | --- | --- | --- | --- | --- | --- | --- |
|  | **NPD** | **LPD** | **e[CO_2_]** | **NPD** | **LPD** | **e[CO_2_]** | **NPD** | **LPD** | **e[CO_2_]** | **NPD** | **LPD** | **e[CO_2_]** | **NPD** | **LPD** | **e[CO_2_]** | **NPD** | **LPD** | **e[CO_2_]** |
| DZ78 | 115 ± 0.7 | 127 ± 0.6 | 127 ± 0.2 | 13 ± 0.9 | 22 ± 0.9 | 16 ± 0.7 | 13 ± 0.9 | 22 ± 0.7 | 16 ± 1.0 | 72 ± 4.2 | 95 ± 5.4 | 81 ± 2.7 | 26 ± 1.8 | 37 ± 2.0 | 31 ± 1.5 | 26.10 ± 0.01 | 27.46 ± 0.16 | 27.61 ± 0.13 |
| SML 242 | 113 ± 0.3 | 102 ± 0.8 | 116 ± 0.2 | 22 ± 1.0 | 32 ± 2.2 | 25 ± 1.4 | 22 ± 1.0 | 32 ± 2.3 | 24 ± 1.2 | 55 ± 1.4 | 70 ± 1.0 | 65 ± 1.9 | 21 ± 2.0 | 41 ± 4.0 | 28 ± 1.2 | 20.81 ± 0.09 | 21.25 ± 0.15 | 21.93 ± 0.06 |
| AI-CHIAO-HONG | 117 ± 0.3 | 122 ± 0.8 | 124 ± 0.2 | 27 ± 2.2 | 44 ± 4.8 | 32 ± 1.2 | 27 ± 2.2 | 44 ± 4.8 | 32 ± 1.1 | 79 ± 4.9 | 107 ± 7.9 | 95 ± 2.0 | 40 ± 3.4 | 47 ± 4.1 | 45 ± 3.1 | 21.45 ± 0.10 | 21.48 ± 0.05 | 21.91 ± 0.07 |
| BYAKKOKU Y 5006 SELN | 112 ± 0.2 | 120 ± 0.8 | 130 ± 0.3 | 17 ± 1.9 | 21 ± 2.3 | 19 ± 1.1 | 17 ± 1.9 | 19 ± 2.5 | 18 ± 1.3 | 77 ± 11.8 | 118 ± 10.7 | 101 ± 3.3 | 36 ± 4.7 | 55 ± 7.8 | 43 ± 2.3 | 18.50 ± 0.02 | 18.62 ± 0.06 | 20.29 ± 0.40 |
| CHAMPA TONG 54 | 136 ± 0.1 | 134 ± 0.7 | 129 ± 0.2 | 10 ± 0.9 | 15 ± 0.9 | 13 ± 1.0 | 10 ± 0.9 | 14 ± 1.4 | 13 ± 1.1 | 53 ± 5.4 | 72 ± 4.1 | 68 ± 1.5 | 26 ± 3.4 | 39 ± 3.9 | 32 ± 2.3 | 30.19 ± 0.14 | 32.17 ± 0.03 | 32.47 ± 0.10 |
| CHINESE | 104 ± 0.7 | 108 ± 0.4 | 106 ± 0.2 | 12 ± 1.6 | 24 ± 2.2 | 19 ± 1.6 | 12 ± 1.6 | 24 ± 2.1 | 18 ± 1.6 | 48 ± 2.8 | 60 ± 4.2 | 57 ± 1.9 | 19 ± 1.7 | 33 ± 4.2 | 26 ± 2.1 | 20.87 ± 0.04 | 21.50 ± 0.01 | 21.60 ± 0.05 |
| DAWEBYAN | 144 ± 0.7 | 131 ± 0.4 | 127 ± 0.1 | 11 ± 1.1 | 18 ± 0.5 | 16 ± 1.3 | 11 ± 1.1 | 18 ± 0.5 | 16 ± 1.3 | 42 ± 6.9 | 59 ± 3.8 | 57 ± 2.9 | 20 ± 4.6 | 29 ± 2.1 | 24 ± 1.7 | 24.06 ± 0.11 | 25.14 ± 0.04 | 25.38 ± 0.01 |
| DJ 123 | 142 ± 0.6 | 123 ± 0.3 | 135 ± 0.2 | 13 ± 1.4 | 24 ± 1.9 | 20 ± 0.9 | 13 ± 1.4 | 24 ± 1.9 | 20 ± 0.9 | 66 ± 5.2 | 106 ± 9.1 | 87 ± 4.2 | 33 ± 1.9 | 43 ± 6.2 | 36 ± 2.1 | 30.02 ± 0.08 | 30.92 ± 0.03 | 31.52 ± 0.09 |
| ECIA76-S89-1 | 80 ± 0.1 | 85 ± 0.5 | 94 ± 0.2 | 12 ± 0.6 | 28 ± 3.5 | 21 ± 1.1 | 12± 0.6 | 26 ± 3.3 | 21 ± 1.1 | 47 ± 3.8 | 75 ± 14.1 | 58 ± 3.0 | 24 ± 1.6 | 32 ± 4.6 | 26 ± 2.2 | 19.84 ± 0.06 | 21.37 ± 0.05 | 22.03 ± 0.05 |
| GHARIB | 108 ± 0.2 | 126 ± 0.2 | 117 ± 0.2 | 14 ± 1.5 | 23 ± 2.7 | 19 ± 1.6 | 14 ± 1.5 | 23 ± 2.7 | 19 ± 1.6 | 68 ± 7.7 | 102 ± 11.6 | 81 ± 5.1 | 32 ± 3.8 | 43 ± 5.4 | 39 ± 3.4 | 19.08 ± 0.09 | 20.49 ± 0.01 | 20.41 ± 0.08 |
| HALWA GOSE RED | 101 ± 0.8 | 103 ± 0.5 | 111 ± 0.7 | 14 ± 1.1 | 21 ± 0.9 | 17 ± 1.4 | 14 ± 1.1 | 20 ± 1.0 | 17 ± 1.4 | 69 ± 3.8 | 109 ± 5.9 | 87 ± 3.5 | 34 ± 1.6 | 54 ± 2.4 | 39 ± 1.6 | 23.23 ± 0.05 | 23.83 ± 0.02 | 24.65 ± 0.08 |
| IRGA 409 | 67 ± 0.5 | 88 ± 0.2 | 80 ± 0.2 | 11 ± 1.2 | 19 ± 2.6 | 16 ± 0.9 | 10 ± 1.3 | 19 ± 2.6 | 16 ± 1.0 | 52 ± 7.5 | 90 ± 5.0 | 72 ± 4.5 | 26 ± 3.7 | 40 ± 3.0 | 35 ± 2.6 | 18.39 ± 0.02 | 18.78 ± 0.02 | 20.03 ± 0.05 |
| JC 117 | 124 ± 0.7 | 113 ± 0.7 | 108 ± 0.5 | 14 ± 0.6 | 25 ± 3.9 | 23 ± 0.6 | 14 ± 0.6 | 25 ± 3.9 | 23 ± 0.6 | 40 ± 4.9 | 80 ± 11.3 | 59 ± 2.8 | 21 ± 1.9 | 37 ± 4.9 | 32 ± 1.7 | 21.14 ± 0.07 | 22.66 ± 0.04 | 22.87 ± 0.04 |
| KARKATI 87 | 121 ± 0.8 | 121 ± 0.5 | 121 ± 0.2 | 13 ± 0.6 | 26 ± 2.0 | 22 ± 2.0 | 13 ± 0.6 | 26 ± 2.0 | 22 ± 2.0 | 71 ± 3.4 | 89 ± 4.4 | 86 ± 2.9 | 37 ± 2.3 | 45 ± 2.7 | 45 ± 2.1 | 22.03 ± 0.01 | 22.32 ± 0.04 | 22.86 ± 0.08 |
| KIANG-CHOU-CHIU | 125 ± 1.1 | 126 ± 0.5 | 111 ± 0.5 | 17 ± 1.0 | 28 ± 2.7 | 24 ± 1.1 | 17 ± 1.0 | 28 ± 2.7 | 24 ± 1.1 | 48 ± 2.4 | 77 ± 9.5 | 61 ± 2.1 | 26 ± 1.6 | 41 ± 3.5 | 33 ± 1.2 | 21.06 ± 0.08 | 21.24 ± 0.02 | 21.53 ± 0.05 |
| MTU9 | 145 ± 0.3 | 140 ± 0.5 | 124 ± 0.6 | 10 ± 0.4 | 19 ± 1.7 | 16 ± 1.0 | 10 ± 0.4 | 19 ± 1.7 | 16 ± 1.0 | 47 ± 3.0 | 86 ± 4.7 | 66 ± 2.6 | 24 ± 1.9 | 40 ± 1.7 | 31 ± 2.7 | 29.35 ± 0.06 | 29.73 ± 0.08 | 29.84 ± 0.07 |
| MUDGO | 120 ± 0.4 | 117 ± 0.1 | 121 ± 0.1 | 10 ± 1.0 | 16 ± 1.2 | 14 ± 0.9 | 10 ± 1.0 | 16 ± 1.2 | 14 ± 0.9 | 57 ± 7.5 | 80 ± 11.1 | 67 ± 2.2 | 24 ± 4.5 | 29 ± 3.4 | 32 ± 1.1 | 26.56 ± 0.03 | 27.22 ± 0.03 | 28.15 ± 0.02 |
| PEH-KUH | 107 ± 0.5 | 104 ± 0.1 | 114 ± 0.7 | 15 ± 0.8 | 25 ± 2.1 | 22 ± 1.1 | 15 ± 0.8 | 25 ± 2.1 | 22 ± 1.1 | 65 ± 2.9 | 98 ± 9.9 | 83 ± 2.8 | 31 ± 3.5 | 44 ± 4.3 | 40 ± 2.3 | 17.07 ± 0.02 | 17.89 ± 0.05 | 17.66 ± 0.07 |
| PTB 30 | 118 ± 0.5 | 122 ± 0.6 | 111 ± 0.6 | 17 ± 1.7 | 35 ± 4.3 | 29 ± 1.3 | 17 ± 1.7 | 35 ± 4.1 | 29 ± 1.3 | 77 ± 8.0 | 112 ± 12.1 | 97 ± 3.7 | 39 ± 4.8 | 53 ± 6.3 | 45 ± 1.5 | 22.96 ± 0.04 | 23.67 ± 0.02 | 23.45 ± 0.06 |
| SURJAMKUHI | 110 ± 2.1 | 121 ± 0.7 | 107 ± 0.7 | 18 ± 2.0 | 34 ± 1.8 | 29 ± 1.1 | 18 ± 2.0 | 34 ± 1.8 | 28 ± 1.0 | 64 ± 10.2 | 112 ± 5.9 | 80 ± 5.1 | 32 ± 4.8 | 45 ± 3.5 | 39 ± 2.5 | 17.55 ± 0.03 | 17.80 ± 0.05 | 18.42 ± 0.06 |
| TIA BURA | 145 ± 0.7 | 123 ± 0.5 | 135 ± 0.5 | 13 ± 1.2 | 20 ± 1.5 | 17 ± 1.2 | 13 ± 1.2 | 20 ± 1.5 | 17 ± 1.2 | 49 ± 5.0 | 61 ± 3.9 | 57 ± 3.3 | 21 ± 2.2 | 27 ± 2.2 | 28 ± 2.1 | 23.26 ± 0.07 | 23.75 ± 0.03 | 23.92 ± 0.06 |
| YODANYA | 115 ± 0.1 | 111 ± 0.6 | 113 ± 0.5 | 24 ± 3.2 | 43 ± 2.4 | 37 ± 1.0 | 24 ± 3.2 | 43 ± 2.4 | 37 ± 0.9 | 72 ± 13.0 | 107 ± 16.5 | 86 ± 3.2 | 36 ± 5.0 | 51 ± 7.2 | 45 ± 2.7 | 22.43 ± 0.06 | 23.40 ± 0.04 | 23.65 ± 0.03 |
| RONDO (4484-1693) | 72 ± 0.1 | 82 ± 0.1 | 86 ± 0.4 | 17 ± 1.5 | 31 ± 0.6 | 25 ± 1.2 | 17 ± 1.5 | 31 ± 1.5 | 25 ± 1.2 | 74 ± 8.4 | 111 ± 3.0 | 95 ± 2.6 | 42 ± 4.7 | 56 ± 1.6 | 51 ± 1.3 | 18.37 ± 0.05 | 19.57 ± 0.02 | 20.36 ± 0.06 |
| LSD (P<0.05) |  | | |  | | |  | | |  | | |  | | |  | | |
| Genotype (G) | 0.89*** | | | 2.84*** | | | 2.82*** | | | 10.45*** | | | 5.45*** | | | 0.12*** | | |
| Treatment (T) | ns | | | 1.03*** | | | 1.02*** | | | 3.77*** | | | 1.97*** | | | 0.05*** | | |
| G x T | 1.54*** | | | 4.92** | | | 4.89** | | | ns | | | ns | | | 0.22*** | | |

^[LSD= Least significant difference; Probability values of the effects of genotypes (G), treatment (T) and their interaction (G xT) for all the traits measured by^ *^ANOVA^* ^; Significance, *** P<0.001; ns= non-significant]^

**Supplemental Table S3.** *ANOVA* for leaf photosynthesis, stomatal conductance and transpiration for experiment III in 2018.

|  | **Leaf photosynthesis (*A)***  (µmol m^-2^ s^-1^) | **Stomatal conductance (*g_s_*)**  (mol m^-2^ s^-1^) | **Transpiration (*E*)**  (mmol m^-2^ s^-1^) | **Night respiration (*R_N_*)**  (µmol m^-2^ s^-1^) | ***R_N_/A* ratio** |
| --- | --- | --- | --- | --- | --- |
| Genotype (G) | 1.66*** | 0.08* | 1.14* | 0.064*** | 0.0036*** |
| Treatment (T) | 1.29*** | 0.06^ns^ | 0.88^ns^ | 0.049*** | 0.0028*** |
| G x T | 2.88*** | 0.13* | 1.97*** | 0.111*** | 0.0063*** |

_[LSD= Least Significant Difference; Significance, ***= P<0.001; **=P<0.01, *= P<0.05]_

**Supplemental Table S4.** List of rice accessions in the *Oryza sativa* subsp. indica diversity panel assembled at IRRI. NA – Not Available.

| **S. No.** | **Acc ID** | **IRGC No.** | **Name** | **Origin** |
| --- | --- | --- | --- | --- |
| 1 | 117600 | IRGC 117600 | AIJIAONANTE | China |
| 2 | 117601 | IRGC 117601 | ARC 10177 | India |
| 3 | 117604 | IRGC 117604 | ASD 1 | India |
| 4 | 117609 | IRGC 117609 | CS-M3 | United States |
| 5 | 117610 | IRGC 117610 | DZ78 | Bangladesh |
| 6 | 117611 | IRGC 117611 | GERDEH | Iran, Islamic Republic Of |
| 7 | 117613 | IRGC 117613 | HABIGANJ BORO 6 | Bangladesh |
| 8 | 117614 | IRGC 117614 | HON CHIM | Hong Kong |
| 9 | 117615 | IRGC 117615 | IAC 25 | Brazil |
| 10 | 117616 | IRGC 117616 | ITALICA CAROLINA | Poland |
| 11 | 117617 | IRGC 117617 | KASALATH | India |
| 12 | 117620 | IRGC 117620 | MEHR | Iran, Islamic Republic Of |
| 13 | 117622 | IRGC 117622 | RTS 14 | Viet Nam |
| 14 | 117623 | IRGC 117623 | BR24 | Bangladesh |
| 15 | 117624 | IRGC 117624 | KIUKI NO. 46 | Japan |
| 16 | 117625 | IRGC 117625 | SML 242 | Suriname |
| 17 | 117630 | IRGC 117630 | 519 | Uruguay |
| 18 | 117631 | IRGC 117631 | 56-122-23 | Thailand |
| 19 | 117632 | IRGC 117632 | 583 | Ecuador |
| 20 | 117635 | IRGC 117635 | AGUSITA | Hungary |
| 21 | 117636 | IRGC 117636 | AI-CHIAO-HONG | China |
| 22 | 117638 | IRGC 117638 | AMPOSTA | Puerto Rico |
| 23 | 117640 | IRGC 117640 | ARC 10086 | India |
| 24 | 117641 | IRGC 117641 | ARC 10352 | India |
| 25 | 117642 | IRGC 117642 | ARC 10376 | India |
| 26 | 117644 | IRGC 117644 | ASWINA 330 | Bangladesh |
| 27 | 117645 | IRGC 117645 | AZERBAIDJANICA | Azerbaijan |
| 28 | 117646 | IRGC 117646 | B 6616 A 4-22-BK-5-4 | United States |
| 29 | 117647 | IRGC 117647 | BABER | India |
| 30 | 117648 | IRGC 117648 | BAGHLANI NANGARHAR | Afghanistan |
| 31 | 117651 | IRGC 117651 | BALDO | Italy |
| 32 | 117652 | IRGC 117652 | BASMATI | Pakistan |
| 33 | 117654 | IRGC 117654 | BELLARDONE | France |
| 34 | 117655 | IRGC 117655 | BENLLOK | Peru |
| 35 | 117657 | IRGC 117657 | BERGREIS | Austria |
| 36 | 117658 | IRGC 117658 | BICO BRANCO | Brazil |
| 37 | 117660 | IRGC 117660 | BISER 1 | Bulgaria |
| 38 | 117662 | IRGC 117662 | BLACK GORA | India |
| 39 | 117665 | IRGC 117665 | BOMBILLA | Spain |
| 40 | 117666 | IRGC 117666 | BOMBON | Spain |
| 41 | 117669 | IRGC 117669 | BUL ZO | Korea, Republic of |
| 42 | 117671 | IRGC 117671 | BYAKKOKU Y 5006 SELN | Australia |
| 43 | 117675 | IRGC 117675 | CANELLA DE FERRO | Brazil |
| 44 | 117680 | IRGC 117680 | CHAMPA TONG 54 | Thailand |
| 45 | 117683 | IRGC 117683 | CHIBICA | Mozambique |
| 46 | 117686 | IRGC 117686 | CHINESE | China |
| 47 | 117687 | IRGC 117687 | CHODONGJI | Korea, Republic of |
| 48 | 117688 | IRGC 117688 | CHUAN 4 | Chinese Taipei (Taiwan) |
| 49 | 117690 | IRGC 117690 | CI 11026 | United States |
| 50 | 117697 | IRGC 117697 | CTG 1516 | Bangladesh |
| 51 | 117698 | IRGC 117698 | CUBA 65 | Cuba |
| 52 | 117699 | IRGC 117699 | CYBONNET | United States |
| 53 | 117700 | IRGC 117700 | DA16 | Bangladesh |
| 54 | 117702 | IRGC 117702 | DARMALI | Nepal |
| 55 | 117703 | IRGC 117703 | DAWEBYAN | Myanmar |
| 56 | 117705 | IRGC 117705 | DEE GEO WOO GEN | Chinese Taipei (Taiwan) |
| 57 | 117706 | IRGC 117706 | DELLA | United States |
| 58 | 117707 | IRGC 117707 | DELREX | United States |
| 59 | 117708 | IRGC 117708 | DEOKJEOKJODO | Korea, Republic of |
| 60 | 117709 | IRGC 117709 | DESVAUXII | Japan |
| 61 | 117710 | IRGC 117710 | DHALA SHAITTA | India |
| 62 | 117711 | IRGC 117711 | DJ 123 | Bangladesh |
| 63 | 117712 | IRGC 117712 | DJ 24 | Bangladesh |
| 64 | 117713 | IRGC 117713 | DJIMORON | Guinea-Bissau |
| 65 | 117715 | IRGC 117715 | DM 43 | Bangladesh |
| 66 | 117717 | IRGC 117717 | DM 59 | Bangladesh |
| 67 | 117718 | IRGC 117718 | DNJ 140 | Bangladesh |
| 68 | 117719 | IRGC 117719 | DOBLE CAROLINA RINALDO BARSANI | Uruguay |
| 69 | 117720 | IRGC 117720 | DOM ZARD | Iran, Islamic Republic Of |
| 70 | 117722 | IRGC 117722 | DOSEL | Spain |
| 71 | 117724 | IRGC 117724 | DV 123 | Bangladesh |
| 72 | 117726 | IRGC 117726 | DZ 193 | Bangladesh |
| 73 | 117727 | IRGC 117727 | EARLY WATARIBUNE | United States |
| 74 | 117728 | IRGC 117728 | ECIA76-S89-1 | Cuba |
| 75 | 117730 | IRGC 117730 | EDOMEN SCENTED | Japan |
| 76 | 117732 | IRGC 117732 | ERYTHROCEROS HOKKAIDO | Poland |
| 77 | 117733 | IRGC 117733 | ESTRELA | Colombia |
| 78 | 117737 | IRGC 117737 | FOSSA AV | Burkina Faso |
| 79 | 117739 | IRGC 117739 | GHARIB | Iran, Islamic Republic Of |
| 80 | 117741 | IRGC 117741 | GHORBHAI | Bangladesh |
| 81 | 117743 | IRGC 117743 | GORIA | Bangladesh |
| 82 | 117744 | IRGC 117744 | GOTAK GATIK | Indonesia |
| 83 | 117745 | IRGC 117745 | GUAN-YIN-TSAN | China |
| 84 | 117746 | IRGC 117746 | GUINEANDAO | NA |
| 85 | 117747 | IRGC 117747 | HALWA GOSE RED | Iraq |
| 86 | 117748 | IRGC 117748 | HATSUNISHIKI | Japan |
| 87 | 117749 | IRGC 117749 | HIDERISIRAZU | Japan |
| 88 | 117754 | IRGC 117754 | I-GEO-TZE | Chinese Taipei (Taiwan) |
| 89 | 117762 | IRGC 117762 | IRAT 44 | Burkina Faso |
| 90 | 117763 | IRGC 117763 | IRGA 409 | Brazil |
| 91 | 117765 | IRGC 117765 | JAMIR | Bangladesh |
| 92 | 117767 | IRGC 117767 | JC 117 | India |
| 93 | 117768 | IRGC 117768 | JC149 | India |
| 94 | 117769 | IRGC 117769 | JHONA 349 | India |
| 95 | 117771 | IRGC 117771 | JOUIKU 393G | Japan |
| 96 | 117772 | IRGC 117772 | KACHILON | Bangladesh |
| 97 | 117773 | IRGC 117773 | KALAMKATI | India |
| 98 | 117774 | IRGC 117774 | KALUBALA VEE | Sri Lanka |
| 99 | 117775 | IRGC 117775 | KAMENOO | Japan |
| 100 | 117776 | IRGC 117776 | KANIRANGA | Indonesia |
| 101 | 117777 | IRGC 117777 | KARABASCHAK | Bulgaria |
| 102 | 117778 | IRGC 117778 | KARKATI 87 | Bangladesh |
| 103 | 117779 | IRGC 117779 | KAUKAU | Mali |
| 104 | 117780 | IRGC 117780 | KAUKKYI ANI | Myanmar |
| 105 | 117781 | IRGC 117781 | KHAO GAEW | Thailand |
| 106 | 117784 | IRGC 117784 | KIANG-CHOU-CHIU | Chinese Taipei (Taiwan) |
| 107 | 117786 | IRGC 117786 | KINASTANO | Philippines |
| 108 | 117788 | IRGC 117788 | KON SUITO | Mongolia |
| 109 | 117789 | IRGC 117789 | KOSHIHIKARI | Japan |
| 110 | 117790 | IRGC 117790 | KOTOBUKI MOCHI | Japan |
| 111 | 117792 | IRGC 117792 | KU 115 | Thailand |
| 112 | 117793 | IRGC 117793 | KUN-MIN-TSIEH-HUNAN | China |
| 113 | 117796 | IRGC 117796 | LAC 23 | Liberia |
| 114 | 117797 | IRGC 117797 | LACROSSE | United States |
| 115 | 117798 | IRGC 117798 | LADY WRIGHT SELN | United States |
| 116 | 117799 | IRGC 117799 | LAMBAYEQUE 1 | Peru |
| 117 | 117803 | IRGC 117803 | LEUANG HAWN | Thailand |
| 118 | 117804 | IRGC 117804 | LIGERITO | Colombia |
| 119 | 117805 | IRGC 117805 | LLANERO 501 | Venezuela |
| 120 | 117806 | IRGC 117806 | LOMELLO | Italy |
| 121 | 117807 | IRGC 117807 | LUK TAKHAR | Afghanistan |
| 122 | 117808 | IRGC 117808 | LUSITANO | Portugal |
| 123 | 117809 | IRGC 117809 | M-202 | United States |
| 124 | 117810 | IRGC 117810 | M. BLATEC | Macedonia |
| 125 | 117811 | IRGC 117811 | MANSAKU | Japan |
| 126 | 117812 | IRGC 117812 | MARATELLI | France |
| 127 | 117813 | IRGC 117813 | MELANOTRIX | Tajikistan |
| 128 | 117815 | IRGC 117815 | MIRITI | Bangladesh |
| 129 | 117817 | IRGC 117817 | MTU9 | India |
| 130 | 117818 | IRGC 117818 | MUDGO | India |
| 131 | 117821 | IRGC 117821 | NIRA | United States |
| 132 | 117824 | IRGC 117824 | NPE 844 | Pakistan |
| 133 | 117826 | IRGC 117826 | O-LUEN-CHEUNG | Chinese Taipei (Taiwan) |
| 134 | 117827 | IRGC 117827 | OKSHITMAYIN | Myanmar |
| 135 | 117828 | IRGC 117828 | ORO | Chile |
| 136 | 117844 | IRGC 117844 | PATNA | Morocco |
| 137 | 117846 | IRGC 117846 | PATO DE GALLINAZO Y 5371 | Australia |
| 138 | 117848 | IRGC 117848 | PEH-KUH | Chinese Taipei (Taiwan) |
| 139 | 117849 | IRGC 117849 | PEH-KUH-TSAO-TU | Chinese Taipei (Taiwan) |
| 140 | 117850 | IRGC 117850 | PHUDUGEY | Bhutan |
| 141 | 117851 | IRGC 117851 | PI 298967-1 | Australia |
| 142 | 117852 | IRGC 117852 | PIRINAE 69 | Yugoslavia |
| 143 | 117853 | IRGC 117853 | PR 304 | Puerto Rico |
| 144 | 117854 | IRGC 117854 | PRATAO | Brazil |
| 145 | 117855 | IRGC 117855 | PRIANO GUAIRA | Brazil |
| 146 | 117856 | IRGC 117856 | PTB 30 | India |
| 147 | 117857 | IRGC 117857 | R 101 | Congo |
| 148 | 117869 | IRGC 117869 | S 4542 A 3-49B-2-12 | United States |
| 149 | 117873 | IRGC 117873 | SADRI TOR MISRI | Iran, Islamic Republic Of |
| 150 | 117876 | IRGC 117876 | SANTHI SUFAID | Pakistan |
| 151 | 117883 | IRGC 117883 | SHIM BALTE | Iraq |
| 152 | 117884 | IRGC 117884 | SHINRIKI | Japan |
| 153 | 117896 | IRGC 117896 | SUFAID | Pakistan |
| 154 | 117897 | IRGC 117897 | SULTANI | Egypt |
| 155 | 117899 | IRGC 117899 | SURJAMKUHI | India |
| 156 | 117900 | IRGC 117900 | SUWEON 362 | Korea, Republic of |
| 157 | 117901 | IRGC 117901 | SZE GUEN ZIM | China |
| 158 | 117902 | IRGC 117902 | T 1 | India |
| 159 | 117903 | IRGC 117903 | T26 | India |
| 160 | 117904 | IRGC 117904 | TA HUNG KU | China |
| 161 | 117905 | IRGC 117905 | TA MAO TSAO | China |
| 162 | 117907 | IRGC 117907 | TAICHUNG NATIVE 1 | Chinese Taipei (Taiwan) |
| 163 | 117914 | IRGC 117914 | TIA BURA | Indonesia |
| 164 | 117917 | IRGC 117917 | TOKYO SHINO MOCHI | Korea, Republic of |
| 165 | 117923 | IRGC 117923 | TROPICAL RICE | Ecuador |
| 166 | 117925 | IRGC 117925 | UZBEKSKIJ 2 | Uzbekistan |
| 167 | 117927 | IRGC 117927 | VARYLA | Madagascar |
| 168 | 117928 | IRGC 117928 | VAVILOVI | Kazakhstan |
| 169 | 117929 | IRGC 117929 | VIALONE | Italy |
| 170 | 117930 | IRGC 117930 | VICTORIA F.A. | Argentina |
| 171 | 117931 | IRGC 117931 | WAB 501-11-5-1 | Côte D'Ivoire |
| 172 | 117937 | IRGC 117937 | WC 521 | China |
| 173 | 117938 | IRGC 117938 | WIR 3764 | Uzbekistan |
| 174 | 117940 | IRGC 117940 | YODANYA | Myanmar |
| 175 | 117943 | IRGC 117943 | ZHE 733 | China |
| 176 | 117944 | IRGC 117944 | ZHENSHAN 2 | China |
| 177 | 121652 | IRGC 121652 | H256-76-1-1-1 | Argentina |
| 178 | 121654 | IRGC 121654 | WC 6 | NA |
| 179 | 124372 | IRGC 124372 | MANZANO | Congo |
| 180 | 124374 | IRGC 124374 | ROMEO | Italy |
| 181 | 125596 | IRGC 125596 | C 101 A 51 | Philippines |
| 182 | 126356 | IRGC 126356 | BEONJO | NA |
| 183 | 126368 | IRGC 126368 | KAW LUYOENG | NA |
| 184 | 126369 | IRGC 126369 | KIBI | NA |
| 185 | 126380 | IRGC 126380 | AGOSTANO | Italy |
| 186 | 126381 | IRGC 126381 | ARIAS | Indonesia |
| 187 | 126382 | IRGC 126382 | EARLY | United States |
| 188 | 126389 | IRGC 126389 | PECOS | United States |
| 189 | 126390 | IRGC 126390 | RONDO (4484-1693) | China |
| 190 | 126392 | IRGC 126392 | RT0034 | United States |
| 191 | 126393 | IRGC 126393 | SABER | United States |
| 192 | 126395 | IRGC 126395 | STEGARU 65 | Romania |
| 193 | 126400 | IRGC 126400 | NSF-TV 34 | NA |
| 194 | 126402 | IRGC 126402 | BENGAL | NA |

References (**Supplementary Figure S12**):

**Sinha PG, Kapoor R, Uprety DC, Bhatnagar AK. 2009.** Impact of elevated CO_2_ concentration on ultrastructure of pericarp and composition of grain in three Triticum species of different ploidy levels. *Environmental & Experimental Botany* **66**: 451-456.

**Sinha PG, Saradhi PP, Uprety DC, Bhatnagar AK. 2011.** Effect of elevated CO_2_ concentration on photosynthesis and flowering in three wheat species belonging to different ploidies. *Agriculture, Ecosystems & Environment* **142**: 432-436.

**Uprety DC, Bisht BS, Dwivedi N, Saxena DC, Mohan R, Raj A, Paswan G, Mitra AP, Garg SC, Tiwari MK et al. 2007.** Comparison between Open Top Chamber (OTC) and Free Air CO_2_ Enrichment (FACE) Technologies to study the response of rice cultivars to elevated CO_2_. *Physiology & Molecular Biology of Plants* **13**: 259-266.
